# Circuits as a simple platform for the emergence of hydrodynamics in deterministic chaotic many-body systems

**DOI:** 10.1038/s41467-026-74457-3

**Published:** 2026-06-30

**Authors:** Sun Woo P. Kim, Friedrich Hübner, Juan P. Garrahan, Benjamin Doyon

**Affiliations:** 1 Department of Physics, King’s College London, London, UK; 2 Department of Mathematics, King’s College London, London, UK; 3 School of Physics and Astronomy, University of Nottingham, Nottingham, UK; 4 Centre for the Mathematics and Theoretical Physics of Quantum Non-Equilibrium Systems, University of Nottingham, Nottingham, UK

**Keywords:** Statistical physics, Fluid dynamics

## Abstract

The emergence of hydrodynamics is one of the deepest phenomena in many-body systems. Arguably, the hydrodynamic equations are also the most important tools for predicting large-scale behaviour. Understanding how such equations emerge from microscopic deterministic dynamics is a century-old problem, despite recent progress in fine-tuned integrable systems. Due to the universality of hydrodynamics, the specific microscopic implementation should not matter. Here, we show that classical deterministic circuits provide a minimal, exact, and efficient platform that admits non-trivial hydrodynamic behaviour for deterministic but chaotic systems. By developing new techniques and focusing on 1D circuits as a proof of concept, we obtain the characteristic dynamics, including relaxation to Gibbs states, exact Euler equations, shocks, diffusion, and exact KPZ super-diffusion. Our methods can be easily generalised to higher dimensions or quantum circuits.

## Abstract (web summary)

The emergence of hydrodynamics in many-body systems is a fundamental phenomenon that has long puzzled physicists, particularly in understanding how macroscopic behavior arises from microscopic deterministic dynamics. This study demonstrates that classical deterministic circuits can serve as a simple platform to study hydrodynamic behavior.

## Introduction

A longstanding approach in physics is to describe the aggregate behaviour of liquids and gases, particulate systems obeying microscopic laws, in terms of effective hydrodynamic equations such as Navier–Stokes^1^. While hydrodynamic equations are one of our best tools for predicting large-scale properties, understanding exactly how they emerge from microscopic interactions is a century-old problem. Hydrodynamics^2,3^ appears to generically emerge in both classical or quantum, deterministic or stochastic systems, whenever there is at least one local conservation law. Crucially, the form of hydrodynamic equations and their phenomenology seem to be in universality classes, independent of specific details of the microscopic dynamics. Is it possible, then, to drastically simplify the microscopic dynamics, thus improving numerical and analytical tractability, and yet obtain all physically relevant, emergent large-scale effects seen in realistic systems?

The key idea behind hydrodynamics is that at large scales of space and time, many-body systems with short-range interactions relax to local equilibrium (thermal, or generalised^4^). This strong assumption is what allows one to predict the hydrodynamic equations in term of equilibrium properties. A way of simplifying the microscopic dynamics is to include a phenomenological stochasticity, such as in the simple exclusion process and its variants^5^. This facilitates the understanding of the emergence of hydrodynamics, as noise directly enables local relaxation by providing a mechanism for losing information. However, the microscopic dynamics of real many-body systems is deterministic. Proving the emergence of hydrodynamics for deterministic systems is part of the famous Hilbert’s Sixth problem, and is still open. This is due to the inherent difficulty in understanding how information that is not lost but instead distributed globally in the form of subtle long-range correlations can account for the appearance of local relaxation. Recently, a highly acclaimed rigorous proof of the Navier–Stokes equation in hard sphere models appeared^6^. While an impressive result, it is only applicable for extremely dilute gases as it uses the Boltzmann equation. In particular, it requires neglecting three-or-more particle scattering. Therefore, a general framework for Hilbert’s Sixth problem for all regimes remains open.

Local relaxation itself is not the full story: a full understanding of diffusive effects, emergent hydrodynamic noise, hydrodynamic long-range correlations, quantum effects, and turbulence remain important questions. For example, it was recently discovered that in the integrable one-dimensional hard sphere model, where analytic computations are possible, long-range correlations dominate at the diffusive order^7^. In chaotic systems, true progress is hindered by the lack of analytically and numerically tractable models. Often, analytically calculating thermal equilibrium is difficult. And even in cases where one can manage to calculate thermal quantities (ex. using the virial expansion), rigorously analysing continuous Hamiltonian dynamics is so intricate that it can put deriving the hydrodynamics beyond reach. Furthermore, numerically checking the predictions of hydrodynamic theories with the required precision is often unattainable. There are only few systems where analytic computations are possible, the most studied of which are integrable systems^4^ (non-chaotic, 1D) and classical anharmonic chains^8^ (1D). However, there exist no such models for chaotic quantum systems or higher-dimensional systems.

In this paper we propose *deterministic* circuit models as a powerful, minimal, exact, and efficient platform to study the emergence of hydrodynamics in chaotic many-body systems. In general, circuit models are defined in discrete space and time with local update rules that can be deterministic or random, classical or quantum. In recent years, there has been great interest in their non-equilibrium behaviour as they combine tractability with truly many-body physics. In the quantum context, random quantum circuits^9–14^ and dual-unitary circuits^15–17^ have become central to understanding the dynamics of entanglement and thermalisation. Quantum circuits are also used to benchmark performance of quantum computers as they can be experimentally realised in existing quantum devices^18^. In turn, classical circuit models are instances of cellular automata^19^, and have been extensively studied in various contexts including pattern formation, Turing-completeness^19^, integrability^20–23^, and dynamical large deviations^24^.

The intersection between circuits and hydrodynamics has been previously explored in the literature, for specific (mostly integrable) examples^23,25^. However, with the exception of integrable models (eg. generalised hydrodynamics of “Rule 54”), this programme has not been carried out explicitly: a proper treatment would involve the evaluation of closed form hydrodynamic equations and verification of their validity. Moreover, our approach here is different in that instead of choosing a specific model and trying to understand its hydrodynamics, we claim that deterministic and chaotic circuit models provide the correct platform to see many kinds of hydrodynamic behaviour.

Circuit models have two major advantages over time-continuous models:

- Classical (and some quantum) circuits are the fastest and simplest systems to simulate numerically, due to their discrete space, time and configurations. System sizes and timescales of order ~ 10^7^ can be simulated, simply out of reach for any other kinds of systems. In addition, quantum circuits can be naturally implemented on quantum computers. These allow for highly precise hydrodynamics.
- Thanks to explicit breaking of time-translation invariance, as we will show, it becomes possible to find many circuit models whose thermal equilibria can be computed analytically and easily in the form of a product states. In particular, one can predict their Euler equation, the leading order of the hydrodynamic description.

While in this paper, as a proof of concept, we restrict to the most simple (and best understood) case of one-dimensional classical systems, our techniques and the above points can be also applied to higher-dimensional and quantum systems.

To substantiate our proposal, we develop methods to systematically deduce the exact conservation laws in any classical deterministic circuit, from which we predict the manifold of Gibbs states, exact thermodynamics and exact Euler-scale hydrodynamic equations. Through an extensive search in the space of circuits, we uncover a panoply of models with non-trivial hydrodynamic effects, which we predict analytically, including the emergence of shocks and Burgers equation^26^, diffusion, and Kardar–Parisi–Zhang (KPZ) super-diffusion^8^. This is the first example of a deterministic, closed system where the Burgers equation emerges. We verify our predictions with large-scale simulations. Notably, it is the first verification in a deterministic system that non-linear fluctuating hydrodynamics predicts the correct functional form of correlations. Further, we develop a novel numerical method, applicable to many-body systems more generally, to visualise the manifold of Gibbs states and establish its dimensionality and relaxation onto it. This provides a proof-of-concept of the direct connection between generic deterministic chaotic dynamics and large-scale hydrodynamics. Importantly, these results demonstrate not only that can circuits be used to obtain analytic hydrodynamic predictions, but also that the explicit breaking of time-translation invariance does not affect the principles of hydrodynamics.

**Fig. 1:**
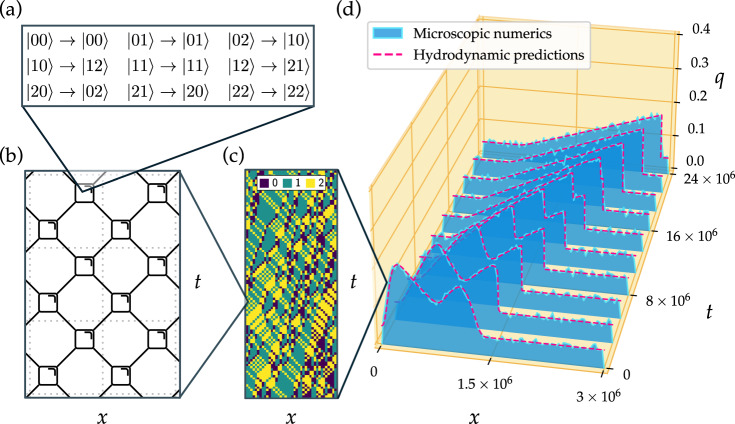
Hydrodynamics of deterministic circuits, illustrated with gate (*d*, *σ*) = (3, 996). **a** Local transition rules. **b** Brickwork structure of the space-time circuit. **c** Example microscopic trajectory. **d** Coarse-grained evolution of the density of the conserved quantity, displaying formation of shocks followed by relaxation to thermal equilibrium. The results from large-scale microscopic numerics, with system size *L* = 3 × 10^6^ and timesteps *T* = 8*L* (blue shading) is accurately captured by the hydrodynamic predictions (red dashed). Here, the numerics is from a single microscopic realisation, followed by averaging of the densities over fluid-cells each of size *ℓ* = 3 × 10^4^. Source data are provided as a Source Data file.

Figure 1 summarises how hydrodynamics emerge: (a) local microscopic dynamical rules, (b) implemented in a spacetime brickwork circuit, (c) give rise to chaotic trajectories, (d) for which we predict the exact large scale behaviour through hydrodynamic theory, including non-trivial phenomenology such as shocks.

## Results

### Classical deterministic circuits

We study classical circuits with deterministic and reversible dynamics in a “brickwork” geometry in 1 + 1 dimensions (i.e., one space and one time dimension). A configuration ***a*** = *a*_1:*L*_ = (*a*_1_, …, *a*_*L*_) of the one-dimensional lattice of *L* sites (with *L* even) is a sequence of local values ${a}_{i}\in {{\mathcal{D}}}$ in a discrete set of *d* states, ${{\mathcal{D}}}=\{0,1,\cdots \,,d-1\}$. We use the notation *i* : *j* to denote support on sites {*i*, *i* + 1, . . , *j*}, and bold symbol to denote support over the whole lattice.

As illustrated in Fig. 1b, at every timestep *τ* = 0, 1, …, *T* − 1, where *T* is the total number of timesteps (also even), we apply local two-body “gates” ${\mathsf{u}}$ in a brickwork fashion with periodic boundary conditions,

1 $${{\mathsf{u}}}_{\tau }:\,{=}{\bigotimes }_{i\in {{{\mathcal{R}}}}_{\tau }}{{\mathsf{u}}}_{i:i+1},$$

where ${{{\mathcal{R}}}}_{\tau }$ is the set of even (resp. odd) sites for even (resp. odd) time *τ*.

A classical, deterministic and reversible gate ${\mathsf{u}}$ is described by an invertible map *σ* on two neighboring sites that maps uniquely a pair of site configurations in ${{\mathcal{D}}}\times {{\mathcal{D}}}$ to another in ${{\mathcal{D}}}\times {{\mathcal{D}}}$. Such a map can be viewed as a permutation $\sigma \in {S}_{{d}^{2}}$, hence there are (*d*^2^)! possible gates. We index each gate by the lexicographic index of its corresponding permutation (“Methods”). We will use *σ* to denote both this index, referring to gates with the notation (*d*, *σ*), and the function that defines the action of the gate, such that given input configurations (*a*, *b*), the output of the gate is *σ*(*a*, *b*) = (*σ*_1_(*a*, *b*), *σ*_2_(*a*, *b*)). For example, the rule of (*d*, *σ*) = (3 996) is shown in Fig. 1a. Throughout, this particular model will be used to illustrate and present our general methodology.

If we denote the local state *a* as a basis vector $\left|a\right\rangle$, then the gate ${\mathsf{u}}$ can be written in bra-ket notation,

2 $${\mathsf{u}}={\sum }_{a,b}\left|{\sigma }_{1}(a,b),{\sigma }_{2}(a,b)\right\rangle \left\langle a,b\right|\,,$$

or equivalently in tensor network notation, 3The evolution operator (1) for odd times then looks as and similarly for the even-time evolution operator.

The dynamics is completely deterministic. However, for the initial state, it is convenient to mimic thermodynamic ensembles, as we are interested in large scales of space and time, where not all microscopic details of the system can be observed or are relevant. Thus, we describe the initial state of the system by a probability vector over configurations, $\left|{p}_{0}\right\rangle={\sum }_{{{\boldsymbol{a}}}}{p}_{0}({{\boldsymbol{a}}})\left|{{\boldsymbol{a}}}\right\rangle$, which evolves over time under the circuit dynamics.

While we restrict our analysis to 1 + 1 dimensions, the generalisation to *D* + 1 dimensions is straightforward. For instance, on the hypercubic lattice of dimension *D*, the local gate would act on the 2^*D*^ sites at the corners of a hypercube. At even times the leftmost sites for the gates are even for each direction, and at odd times they are odd. There are $\left({d}^{{2}^{D}}\right)!$ such gates for *d* local states.

We would like to note that a subset of these models are also studied in a recent work, ref. ^27^ (which appeared after the first version of our paper was made public), focusing mostly on a complementary set of questions. Among other results, ref. ^27^ identifies gates with other types of anomalous transport and gates with extensive conserved quantities (see below) with quasi-local densities. This further validates the richness of these simple systems.

### Conserved quantities (and how to find them)

An important characteristic of a dynamical system with local interactions is the set of extensive *conserved quantities* (CQs) that it admits. For deterministic classical circuits considered here, these are real functions of configurations *F*(***a***) invariant under time evolution, *F*(**u**_*n*_ ⋯ **u**_1_***a***) = *F*(***a***) for some *n*. Most CQs can be decomposed as *F* = ∑_*i*_*F*_*i*_, where the *local densityF*_*i*_ depends only on configurations in a finite radius, which we define as its “locality”, around site *i*. *F*_*i*_ satisfies a microscopic continuity equation with an associated local current in discrete space-time. Such continuity equations control the large-scale dynamics, as at large scales they become continuous conservation laws at the basis of hydrodynamics.

The search for CQs is fundamental to our results. For practical reason, we restrict our search to densities up to a given locality. To predict the correct hydrodynamic equations, however, one must rule out further CQs, including those with *quasi-local densities* present in some models^28–31^. We do this by using our numerical algorithm that uncovers the manifold of Gibbs states, discussed in the next section, as it intrinsically accounts for *the complete set of CQs*.

Naively, one could construct a CQ at the gate level by associating a local “energy” *f*(*a*) to each state, then demand that the gate conserves it for any pair of inputs. This corresponds to the condition

4 $$\left(\left\langle f\right|\left\langle -\right|+\left\langle -\right|\left\langle f\right|\right){\mathsf{u}}=\left\langle f\right|\left\langle -\right|+\left\langle -\right|\left\langle f\right|,$$

where $\left\langle f\right|={\sum }_{a}\,f(a)\left\langle a\right|$ and we have introduced the “flat” state $\left\langle -\right\vert={\sum }_{a}\left\langle a\right\vert=\intercal$, which, as the gates are permutations, is always a CQ (indicating probability conservation).

In general, due to the brickwork structure of the circuit, local densities starting on even and odd sites, $\left\langle \,{f}^{{{\rm{o}}}}\right|$ and $\left\langle \,{f}^{{{\rm{e}}}}\right|$, can differ. Moreover, the CQ may be suported on (2*l* − 1) sites, only be conserved after *n* timesteps of evolution, and periodic with *m* spatial translations. We will refer to *n* and *m* as the temporal and spatial periodicities of the CQ, respectively. We develop a procedure to determine CQs up to locality (2*l* − 1), which only requires a solution to the local linear equation for $\left\langle \,{f}^{{{\rm{o}}}}\right|\oplus \left\langle \,{f}^{{{\rm{e}}}}\right|$ (“Methods”).

When *l* = *m* = *n* = 1 we have a *simple CQ* (SCQ), that is, a single-site CQ with spatial and temporal periodicity 1. For SCQs, the linear equation for $\left\langle \,{f}^{{{\rm{o}}}}\right|\oplus \left\langle \,{f}^{{{\rm{e}}}}\right|$ is 5For example, solving Eq. (5) for (*d*, *σ*) = (3996), we find the SCQ

6 $$\left\langle {f}_{{{\rm{e}}}}\right|=\left\langle 1\right|,\,\left\langle {f}_{{{\rm{o}}}}\right|=-\left\langle 0\right|\,,$$

meaning that the number of $\left|1\right\rangle$ s on even sites minus the number of $\left|0\right\rangle$ s on odd sites is conserved. We find no other CQs up to *l*, *n*, *m* ≤ 5 for this circuit.

The simplest circuits one could have considered are those with *d* = 2. However, an exhaustive search over all (2^2^)! = 24 gates using above methods find that they either have zero, or appear to have a tower of CQs; see Supplementary Sec. A. In the former case, we expect thermalisation to the infinite temperature state and no hydrodynamics. The latter case is likely to be integrable: these are described by the theory of generalised hydrodynamics^32–35^ and are known to have non-generic hydrodynamics (e.g., they do not admit shock formation^36–38^). They will not be discussed here. We will consider circuits with local dimension *d* = 3, focusing on gates with a non-zero but finite number of CQs in order to study the full range of generic hydrodynamic phenomenology.

### Manifold of stationary states

With the previous method, we can exactly determine CQs up to given locality and periodicities, but cannot rule out CQs with higher localities and periodicities nor the larger class of CQs with quasi-local densities. Here, we describe an independent numerical method that can determine their number by studying stationary states of the dynamics.

Following the general principles of many-body physics, we expect that any initial state will relax in such a way that averages of local observables will be given by those of “thermal states” (maximal entropy states) with Gibbs probability

7 $$\left|{p}_{\vec{\beta }}\right\rangle=\frac{1}{{{\mathcal{Z}}}(\vec{\beta })}\,{\sum }_{{{\boldsymbol{a}}}}{e}^{-\vec{\beta }\cdot \vec{F}({{\boldsymbol{a}}})}\left|{{\boldsymbol{a}}}\right\rangle \,.$$

Here, $\vec{F}({{\boldsymbol{a}}})={(\left\langle {F}^{(\alpha )}| {{\boldsymbol{a}}}\right\rangle )}_{\alpha=1:{N}_{{{\rm{cq}}}}}$ is the set of CQs, $\vec{\beta }={({\beta }_{\alpha })}_{\alpha=1:{N}_{{{\rm{cq}}}}}$ is the set of conjugate parameters (or Lagrange multipliers), *N*_cq_ is the total number of CQs. We use the arrow notation to indicate a vector in CQ-species space. Since Eq. (7) only has *N*_cq_ degrees of freedom, if we plot the thermalised values of *M* > *N*_cq_ observables, *X*^(1)^, *X*^(2)^, …, *X*^(*M*)^ starting from different initial conditions, we should find that they lie on the *N*_cq_-dimensional submanifold $({\left\langle {X}^{(1)}\right\rangle }_{\vec{\beta }},{\left\langle {X}^{(2)}\right\rangle }_{\vec{\beta }},\ldots,{\left\langle {X}^{(M)}\right\rangle }_{\vec{\beta }})$ spanned by $\vec{\beta }$. Here, thermal expectations ${\langle \cdot \rangle }_{\vec{\beta }}$ are given by

8 $${\left\langle X\right\rangle }_{\vec{\beta }}: \,={\sum }_{{{\boldsymbol{a}}}}X({{\boldsymbol{a}}})\,{e}^{-\vec{\beta }\cdot \vec{F}({{\boldsymbol{a}}})}/{{\mathcal{Z}}}(\vec{\beta })=\left\langle X| {p}_{\vec{\beta }}\right\rangle \,.$$

**Fig. 2:**
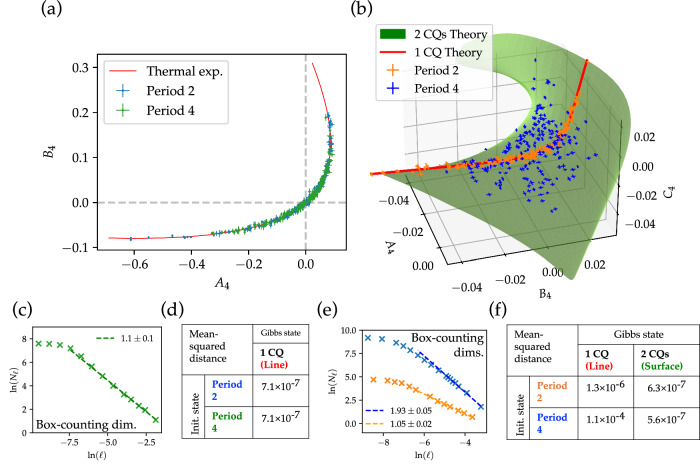
Expectation values of observables embedded in low-dimensional manifolds. Randomly generated observables of random initial states overlaid on theoretical thermal expectation values, for *β* ∈ [ − 5, 5] for *L* = 20000, *T* = 1000, averaged over the last 10 timesteps of multiple 4 (“Methods”). The error bars are the standard errors obtained from this averaging. **a** For gate (*d*, *σ*) = (3, 996), we expect only a single conserved quantity (CQ). For both kinds of initial states with spatial periodicities *m* = 2, 4, we see good evidence that the observables lie on a one-dimensional manifold, and that their values agree with the thermal expectations. **b** For gate (*d*, *σ*) = (3, 229177), we expect two conserved quantities, one with *m*_1_ = 2 and another “staggered” quantity with *m*_2_ = 4. For initial states with *m* = 2, the data lies on a one-dimensional curve corresponding to thermal expectation on only the first CQ, as it does not couple to the staggered CQ. Using instead initial states with *m* = 4, we find that data lies on a two-dimensional surface of thermal expectation on both CQs. Note that the crosses denote standard error. **c**, **e** Box-counting dimensions extracted from the generated points by fitting a line to box size *ℓ* and number of filled boxes *N*_*ℓ*_, agreeing with expected number of conserved quantities. **d**, **f** Mean-squared distance between the generated points and manifolds. Note the 3 orders of magnitude difference for mean-squared distance with 1 CQ (Line) and 2 CQ (Surface) theories for period 4 initial states. For visibility, only 200 points are shown in (**a**) and (**b**) but 2000 points are used to extract box-counting dimensions in (**c**), (**e**). Source data are provided as a Source Data file.

For instance, if *N*_cq_ = 1, the plot should converge to a one-dimensional curve. This is illustrated for gate (*d*, *σ*) = (3 996) in Fig. 2a. On the other hand, if *N*_cq_ = 2, it will be two-dimensional, shown in Fig. 2b for circuit (*d*, *σ*) = (3, 229117). If the plot agrees with the predicted *N*_cq_-dimensional manifold, we have the full set of CQs, and in particular, rule out the existence of CQs with quasi-local densities, which we demonstrate for the first time to contribute to the manifold of Gibbs states, in Supplementary Fig. 1. This analysis can be made quantitative by verifying the box-counting dimension of the manifold (Fig. 2c, e) and, in the case where prediction for Gibbs states is available, the mean-squared displacement to the theoretical predictions (Fig. 2d, f). For further details and generalisation of the method, see “Methods”.

Using this method, we confirm the existence of gates with only one CQ that is a SCQ, such as (*d*, *σ*) = (3 996). Such gates are simplest examples which admit non-trivial phenomenology, and we define them as *simple gates* (SGs). For SGs, the thermal state factorises into a product state, $\left|{p}_{\beta }\right\rangle=\cdot \cdot \left|{p}_{\beta }^{{{\rm{e}}}}\right\rangle \left|{p}_{\beta }^{{{\rm{o}}}}\right\rangle \cdot \cdot$, where $\left|{p}_{\beta }^{{{\rm{e}}},{{\rm{o}}}}\right\rangle=(1/{z}_{\beta }^{{{\rm{e}}},{{\rm{o}}}}){\sum }_{a}\exp [-\beta {f}^{{{\rm{e}}},{{\rm{o}}}}(a)]\left|a\right\rangle$, with ${z}_{\beta }^{{{\rm{e}}},{{\rm{o}}}}={\sum }_{a}\exp [-\beta {f}^{{{\rm{e}}},{{\rm{o}}}}(a)]$. The action of SGs on their thermal states is 9In general, for CQs with larger support, Eq. (7) corresponds to a matrix product state.

### Constructing the hydrodynamic equations

Next, we derive the macroscopic hydrodynamic equations. These are emergent PDEs for coordinates *x* = *i* and *t* = *τ*, whose discrete increments vanish with respect to the large scale ~ *L* of variations. For convenience, we take (*x*, *t*) as continuous variables interpolating between space-time lattice points of the circuit. If *L* is very large, it is natural to assume that the state is in local equilibrium, with $\vec{\beta }(x,t)$ varying on the scale ~ *L*. In order to get the hydrodynamic equations, we first determine the microscopic continuity equations for local charges $\left\langle \vec{q}\right|={\left(\left\langle {q}^{(\alpha )}\right|\right)}_{\alpha \in 1:{N}_{{{\rm{cq}}}}}$ and current $\left\langle \vec{{{\rm{J}}}}\right|={\left(\left\langle {{{\rm{J}}}}^{(\alpha )}\right|\right)}_{\alpha \in 1:{N}_{{{\rm{cq}}}}}$ (“Methods”). Then, we specialise the charges and currents to their averages in the local Gibbs states $\vec{\beta }(x,t)$, that is, $\vec{q}(x,t):={\left\langle \vec{q}\right\rangle }_{\vec{\beta }(x,t)}$ and $\vec{{{\rm{J}}}}(x,t):={\left\langle \vec{{{\rm{J}}}}\right\rangle }_{\vec{\beta }(x,t)}$, cf. Eq. (8). By inverting the first relation, which is guaranteed to be possible by the convexity of free energy, we can write the currents as functions of the charges $\vec{{{\rm{J}}}}(\vec{q}(x,t))$ and deduce the Euler-scale hydrodynamics equation

10 $${\partial }_{t}\vec{q}(x,t)=-{\partial }_{x}\vec{{{\rm{J}}}}(x,t)=-\overleftrightarrow{A}{\partial }_{x}\vec{q}(x,t)\,.$$

Here, ${[\overleftrightarrow{A}]}_{\alpha,{\alpha }^{{\prime} }}=\partial {{{\rm{J}}}}^{(\alpha )}/\partial {q}^{({\alpha }^{{\prime} })}$ is the “flux Jacobian”.

For SGs,

11 $${\left\langle {{\rm{J}}}\right\rangle }_{\beta }={\left\langle f\right\rangle }_{{{\rm{o}}},\beta }-{\left\langle f\right\rangle }_{{{\rm{e}}},\beta },$$

i.e., the average current is zero when the local density is the same on even and odd sites (Supplementary Sec. C). Therefore, the naive construction Eq. (4) does not allow for non-zero currents.

**Fig. 3:**
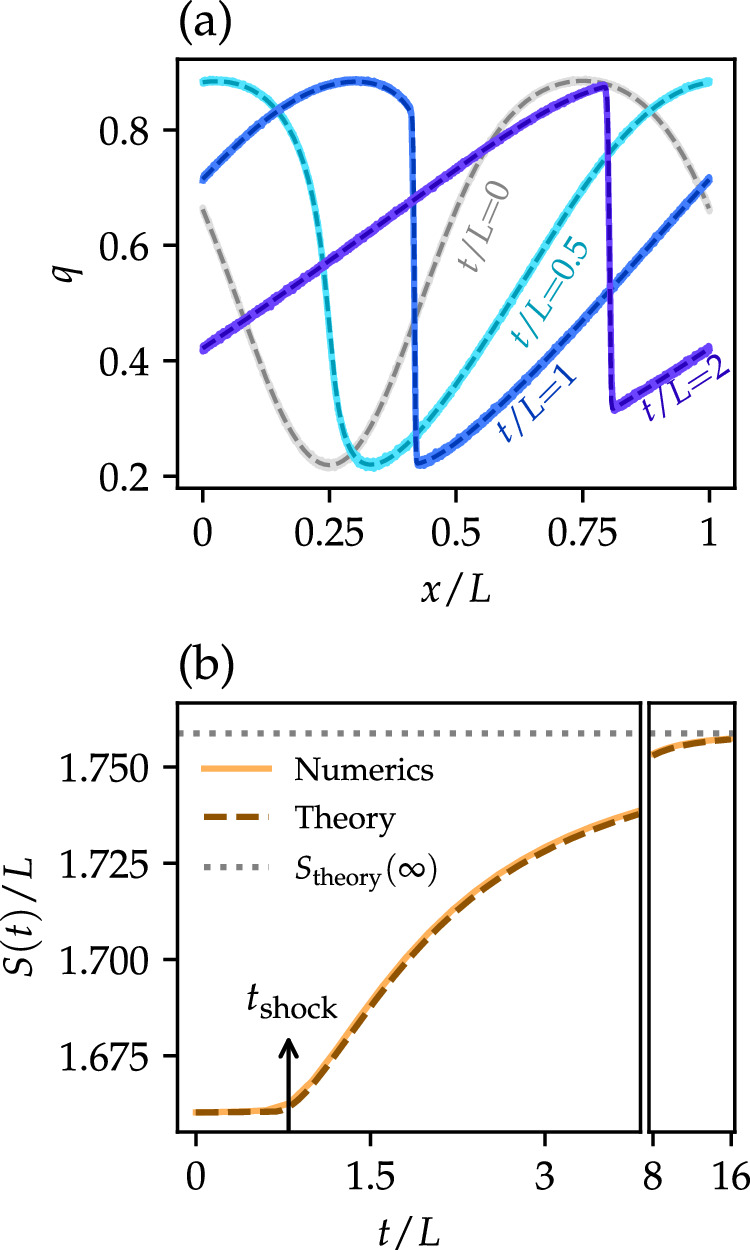
Rapid thermalisation from formation of shocks in deterministic circuits, illustrated with gate (*d*, *σ*) = (3, 996). **a** Formation of shocks. Microscopic numerics for *L* = 10^5^ averaged over 10^5^ samples (solid lines, linewidth corresponds to standard deviation) verifies weak hydrodynamic solutions (dashed lines) given by Rankine–Hugoniot conditions. **b** Entropy production from shocks. *S*(*t*) is constant until shock formation time *t*_shock_ ≈ 0.95*L*, which facilitates entropy production until saturating to a theoretical maximum value *S*_theory_(*∞*) ≈ 1.758*L* at *t*_eq_ ≈ 16*L*. Here, *L* = 8 × 10^4^ and the result is averaged over 10^3^ samples. Source data are provided as a Source Data file.

For (*d*, *σ*) = (3996), we find the thermal charge and currents

12 $${\left\langle q\right\rangle }_{\beta }=\frac{1}{2{e}^{\beta }+1}+\frac{2}{{e}^{\beta }+2}-1,$$

13 $${\left\langle {{\rm{J}}}\right\rangle }_{\beta }=\frac{1}{-2{e}^{\beta }-1}+\frac{2}{{e}^{\beta }+2}-1,$$

resulting in ${{\rm{J}}}(q)=\frac{1}{3}\left(2-\sqrt{9{q}^{2}+16}\right)$ and a simple Euler-scale hydrodynamic equation,

14 $${\partial }_{t}q(x)=-v(q(x)){\partial }_{x}q(x),$$

where the velocity is given by $v(q)=-3q/\sqrt{16+9{q}^{2}}$. In Fig. 3a, we show excellent agreement between Eq. (14) and the microscopic numerics for the circuit. Here, the evolution is deterministic. To obtain observables from distributions of initial states, we average observables over multiple samples. Alternatively, a single sample can be coarse-grained, see Fig. 1d, demonstrating that large-scale effects are self-averaging.

With the change of variables *ρ* = *v*(*q*), Eq. (14) can be mapped to the Burgers equation for *ρ*,

15 $${\partial }_{t}\rho (x,t)+\rho (x,t){\partial }_{x}\rho (x,t)=0.$$

Note that it is impossible to obtain Burgers equation as the hydrodynamics of time-continuous Hamiltonian systems: In 1D Hamiltonian systems, the Hamiltonian is always conserved, and the energy current in a Gibbs state vanishes^39^. Hence, if only the Hamiltonian is conserved, the Euler hydrodynamic equations are trivial ∂_*t*_*q* = 0. The only 1D Hamiltonian systems that have a non-trivial hydrodynamics are those with multiple CQs (implying that the hydrodynamic equations are multiple coupled equations). This restriction does not apply to circuits since they break continuous time-translation symmetry and thus have no conserved Hamiltonian. To the best of our knowledge, the circuits here are the first examples of deterministic and closed microscopic models that give rise to the Burgers equation.

### Shocks, entropy and thermalisation

When discontinuities are absent, Euler-scale hydrodynamic equations conserve the total entropy of the system *S* = ∫d*xs*(*q*(*x*)), with a local conservation law for the entropy density *s*(*q*(*x*)), see ref. ^34^. However, generically, Euler-scale equations develop shocks after finite time^26^. This is illustrated Fig. 1d for the hydrodynamic Eq. (14) of gate (*d*, *σ*) = (3 996). Shocks are discontinuities in density profiles where entropy is not conserved. The concave entropy density *s*(*q*) is given by

16 $$s(q): \, = {\left\langle -{{\rm{ln}}}p\right\rangle }_{\beta (q)}.$$

One may evaluate the shock formation time *t*_shock_ as

17 $${t}_{{{\rm{shock}}}}={\left({\sup }_{x}{v}^{{\prime} }({q}_{0}(x)){\partial }_{x}{q}_{0}(x)\right)}^{-1},$$

where *q*_0_(*x*) is the initial condition for the charge profile. This time is given by the first time characteristics cross. It can be derived analogously to the shock time for Burger’s equation^40^.

The dynamics of shocks cannot be uniquely predicted without additional information put in by hand (“Methods”). On physical grounds, imposing charge conservation, the velocity of shocks *v*_shock_ is expected to be given by the Rankine–Hugoniot condition^26^,

18 $${v}_{{{\rm{shock}}}}=\Delta {{\rm{J}}}/\Delta q,$$

where for any function *h*(*x*), $\Delta h=h({x}_{{{\rm{shock}}}}^{+})-h({x}_{{{\rm{shock}}}}^{-})$ is its jump across the shock. A single shock produces entropy

19 $$\frac{{{\rm{d}}}}{{{\rm{d}}}t}S=\Delta g-{v}_{{{\rm{shock}}}}\Delta s\ge 0,$$

which is always positive if ${v}^{{\prime} }(q)\ge 0$, and where *g*(*q*) is the entropy current, with ${g}^{{\prime} }(q)={s}^{{\prime} }(q)v(q)$. Once the shock appears, entropy increases until equilibration: global relaxation from inhomogeneous initial condition occurs on Euler timescale. Note that this is markedly different from integrable systems and more generally “linearly degenerate” systems, where the absence of shocks implies entropy conservation on the Euler-scale resulting in significantly longer thermalisation timescales.

The above predictions can be explicitly verified for classical deterministic circuits. We show this in Fig. 3 for (*d*, *σ*) = (3 996): in (a) the charge profiles from hydrodynamic predictions of Eqs. (17) and (18) (dashed lines, “Methods”) are compared with those from the microscopic dynamics (solid lines) for an initial profile *q*_0_(*x*) (grey line). Note that hydrodynamics is still accurate even after shock formation. Moreover, *s*(*q*) can be explicitly evaluated as

20 $$s(q)=q{{\rm{ln}}}\frac{5q+\sqrt{16+9{q}^{2}}}{4(1+q)}+{{\rm{ln}}}\frac{16-9q+5\sqrt{16+9{q}^{2}}}{(1+q)(-5+\sqrt{16+9{q}^{2}})}.$$

Combining with hydrodynamic predictions, we compare entropy predictions from hydrodynamics with microscopic numerics in Fig. 3b to excellent agreement. This explicitly confirms predictions of the Rankine–Hugoniot conditions.

### Kardar–Parisi–Zhang superdiffusion from non-linear fluctuating hydrodynamics

The hydrodynamic description enables us to not only describe one-point averages, but also two and higher order correlation functions. It is generally expected that in one dimension two-point correlators scale anomalously, instead of diffusively. The exceptions are 1D linearly degenerate systems^7^, where they are diffusive. These anomalous scalings are explained by non-linear fluctuating hydrodynamics (NLFH)^8^, an effective theory which assumes emergent diffusion and hydrodynamic noise based on physical grounds. Note that this is distinct from microscopic noise which is not present in our circuits. In general, NLFH results in systems of coupled KPZ equations. While it is possible to extract their scaling exponents, so far, their asymptotic shapes could only be computed approximately using mode coupling theory^41–43^. Again, the situation is significantly simpler in our gates, whose non-linear fluctuating hydrodynamics maps exactly to a single KPZ equation (see Supplementary Sec. E for a derivation). This allows us to not only predict KPZ 2/3 scaling of the two-point correlation function, but also its exact asymptotic shape and superdiffusion constant *λ*_B_.

Expanding Eq. (10) around a constant background charge, *q*(*x*, *t*) = *q*_0_ + *δq*(*x*, *t*), where ${q}_{0}={\left\langle q\right\rangle }_{\beta }$, we have

21 $${\partial }_{t}\delta q=-{\partial }_{x}\left(v\delta q+\frac{1}{2}{{{\rm{J}}}}^{{\prime\prime} }({q}_{0})\delta {q}^{2}+\cdots \right),$$

where *v* = *v*(*q*_0_). If J^*″*^(*q*_0_) ≠ 0, NLFH predicts that the correlation function ${\left\langle q(x,t)q(0,0)\right\rangle }_{\beta }^{{{\rm{c}}}}$ will be KPZ superdiffusive,

22 $${\left\langle {q}(x,t)q(0,0)\right\rangle }_{\beta }^{{{\rm{c}}}}=\frac{C}{{({\lambda }_{{{\rm{B}}}}t)}^{2/3}}\,{f}_{{{\rm{KPZ}}}}\left(\frac{x-vt}{{({\lambda }_{{{\rm{B}}}}t)}^{2/3}}\right).$$

Here $C=\int \,dx{\left\langle q(x,0)q(0,0)\right\rangle }_{\beta }^{{{\rm{c}}}}=\frac{1}{2}\,{{\rm{var}}}{[{q}_{1:2}]}_{\beta }$, *f*_KPZ_ is the universal KPZ scaling function, and the superdiffusion constant is (Supplementary Sec. E)

23 $${\lambda }_{{{\rm{B}}}}=\sqrt{2C}| {{{\rm{J}}}}^{{\prime\prime} }({q}_{0})| .$$

**Fig. 4:**
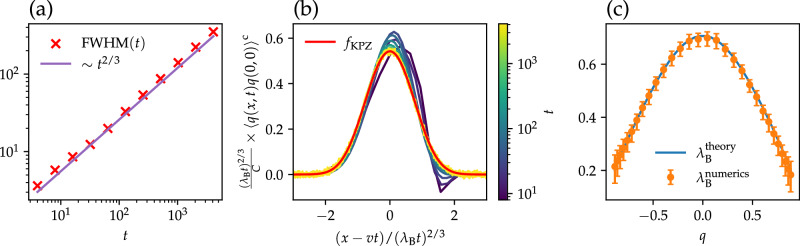
KPZ super-diffusion in deterministic circuits with one conserved quantity, illustrated with gate (*d*, *σ*) = (3, 996). **a** Super-diffusive scaling of the full-width half maximum (FWHM) of the correlation function ${\langle q(x,t)q(0,0)\rangle }_{\beta=0}^{{{\rm{c}}}}$ obtained from microscopic numerics (red crosses, *L* = 2^14^ and averaged over 10^6^ samples), consistent with *t*^2/3^ scaling (purple line). **b** Data collapse of ${\langle q(x,t)q(0,0)\rangle }_{\beta=0}^{{{\rm{c}}}}$, which also converges to the universal KPZ function *f*_KPZ_ (red line). **c** Theoretical prediction of the super-diffusive constant *λ*_B_ (blue line) compared to the value extracted from the numerics (orange markers, *L* = 10^3^ and averaged over 10^6^ samples). This value was extracted from the height of the correlation function at the origin (errorbars represent the standard deviation of this fit). Source data are provided as a Source Data file.

In Fig. 4, we compare theoretical predictions to microscopic numerics for (*d*, *σ*) = (3 996) to excellent agreement. To best of our knowledge, this is the first verification that non-linear fluctuating hydrodynamics predicts the correct asymptotic shape of the correlation function in a deterministic system. Furthermore, the validity of NLFH demonstrates the observation of hydrodynamic noise on large scales, even in microscopically deterministic models.

Note that gates with ∣J^*″*^(q^*^)∣ = 0 for some q^*^ can host both diffusive and superdiffusion correlations. At q^*^ the superdiffusion constant goes to zero and we predict diffusive correlations. For example, the SG (*d*, *σ*) = (3, 1092), with rules

24 $$	 \left|00\right\rangle \to \left|00\right\rangle \,\left|01\right\rangle \to \left|10\right\rangle \,\left|02\right\rangle \to \left|20\right\rangle \\ 	 \left|10\right\rangle \to \left|01\right\rangle \,\left|11\right\rangle \to \left|11\right\rangle \,\left|12\right\rangle \to \left|12\right\rangle \\ 	 \left|20\right\rangle \to \left|02\right\rangle \,\left|21\right\rangle \to \left|21\right\rangle \,\left|22\right\rangle \to \left|22\right\rangle $$

and SCQ $\left\langle {f}_{{{\rm{e}}}}\right|=-\frac{1}{2}\left\langle 0\right|$, $\left\langle {f}_{{{\rm{o}}}}\right|=\frac{1}{2}\left\langle 1\right|+\left\langle 2\right|$, has diffusive correlations at ${q}^{*}=\sqrt{3}\sin (\pi /9)$, and superdiffusive correlations elsewhere, see Fig. 5. More precisely, if ∣*q* − q^*^∣ ~ *δ* is small but finite, then diffusive phenomenology will only be a transient effect until times of order ~ 1/*δ*^4^, after which it is overtaken by KPZ superdiffusion. This simple estimate is obtained by equating the diffusive width ~ *t*^1/2^ with the super-diffusive width ${({\lambda }_{{{\rm{B}}}}t)}^{2/3} \sim {(\delta \times t)}^{2/3}$.

**Fig. 5:**
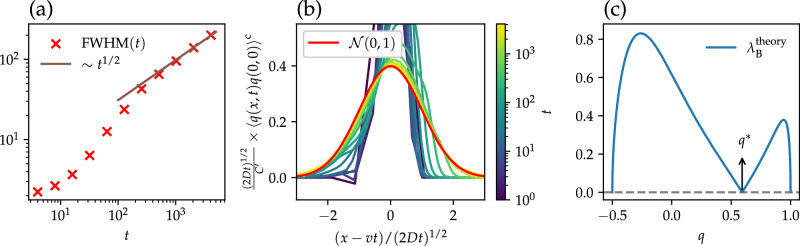
Coexistence of diffusion and KPZ superdiffusion in deterministic circuits, illustrated with gate (*d*, *σ*) = (3, 1092). At special point ${q}^{*}=\sqrt{3}\sin (\pi /9)$, **a** the correlations show diffusive *t*^1/2^ scaling, and **b** converge to a standard normal ${{\mathcal{N}}}(0,1)$ when suitably scaled with the diffusion, *D* = 0.91690(1), and height, ${C}^{{\prime} }=0.17840(1)$, constants. **c** Away from q^*^, the correlations show superdiffusion. Source data are provided as a Source Data file.

### Other hydrodynamic phenomenology

While we focused our method on a handful of circuits in the above as a proof-of-concept, our search of *d* = 3 gates reveals a wealth of other hydrodynamic phenomenology, demonstrating the potential of these deterministic circuits as a platform to study many kinds of hydrodynamics. We discuss representative gates of some below.

• **Staggered conserved quantities**. Gates with CQs with nontrivial periodicities *m*, *n* > 1 exist. An example is (*d*, *σ*) = (3, 229117), with rules

25 $$	 \left|00\right\rangle \to \left|12\right\rangle \,\left|01\right\rangle \to \left|22\right\rangle \,\left|02\right\rangle \to \left|20\right\rangle \\ 	 \left|10\right\rangle \to \left|02\right\rangle \,\left|11\right\rangle \to \left|00\right\rangle \,\left|12\right\rangle \to \left|10\right\rangle \\ 	 \left|20\right\rangle \to \left|01\right\rangle \,\left|21\right\rangle \to \left|11\right\rangle \,\left|22\right\rangle \to \left|21\right\rangle \,.$$

Here, there is one SCQ,

26 $$\left\langle {f}_{{{\rm{e}}}}^{(1)}\right|=\left\langle 1\right|+\left\langle 2\right|,\,\left\langle {f}_{{{\rm{o}}}}^{(1)}\right|=\left\langle 0\right|+2\left\langle 1\right|+\left\langle 2\right|\,,$$

and a second CQ with *μ*_2_ = *λ*_2_ = *e*^*iπ*/2^,

27 $$\left\langle {f}_{{{\rm{e}}}}^{(2)}\right|=\left\langle 0\right|-\left\langle 1\right|+\left\langle 2\right|,\,\left\langle {f}_{{{\rm{o}}}}^{(2)}\right|=\left\langle 1\right|+2\left\langle 2\right|\,,$$

meaning it is conserved only after *n*_2_ = 4 timesteps and is periodic over *m*_2_ = 4 translations. To confirm that there are only two CQs in this circuit, we choose three observables (*A*_4_, *B*_4_, *C*_4_) on randomly sampled initial states, and plot their thermal expectation values. As shown in Fig. 2b, these values lie on a two-dimensional surface as predicted by theory, Eq. (7). Since Eq. (27) changes sign every two sites, if the sampled initial states are spatially period-2, the hydrodynamics decouples (see Supplementary Sec. D for details) and the data falls on a one-dimensional curve, corresponding to the *β*_2_ = 0 predictions. In comparison, for (*d*, *σ*) = (3 996), both period-2 and period-4 initial states lie on a one-dimensional curve, as Fig. 2a.

• **Fermi-Pasta-Ulam-Tsingou (FPUT) phenomenology**. The SCQ of SG (*d*, *σ*) = (3, 2312) with rules

28 $$	 \left|00\right\rangle \to \left|00\right\rangle \,\left|01\right\rangle \to \left|01\right\rangle \,\left|02\right\rangle \to \left|12\right\rangle \\ 	 \left|10\right\rangle \to \left|10\right\rangle \,\left|11\right\rangle \to \left|11\right\rangle \,\left|12\right\rangle \to \left|20\right\rangle \\ 	 \left|20\right\rangle \to \left|21\right\rangle \,\left|21\right\rangle \to \left|02\right\rangle \,\left|22\right\rangle \to \left|22\right\rangle,$$

is $\left\langle {f}_{{{\rm{e}}},{{\rm{o}}}}\right|=\left\langle 2\right|$, i.e., it conserved the number of $\left|2\right\rangle$ s. Eq. (11) predicts J(*q*) = J^*″*^(*q*) = 0 and therefore diffusive correlations. However, at low densities, precluding this diffusive effect, there is a transient state that is characteristic of integrability. It is similar to the FPUT phenomenology typically observed in one-dimensional systems with energy and momentum conservation^44^.

**Fig. 6:**
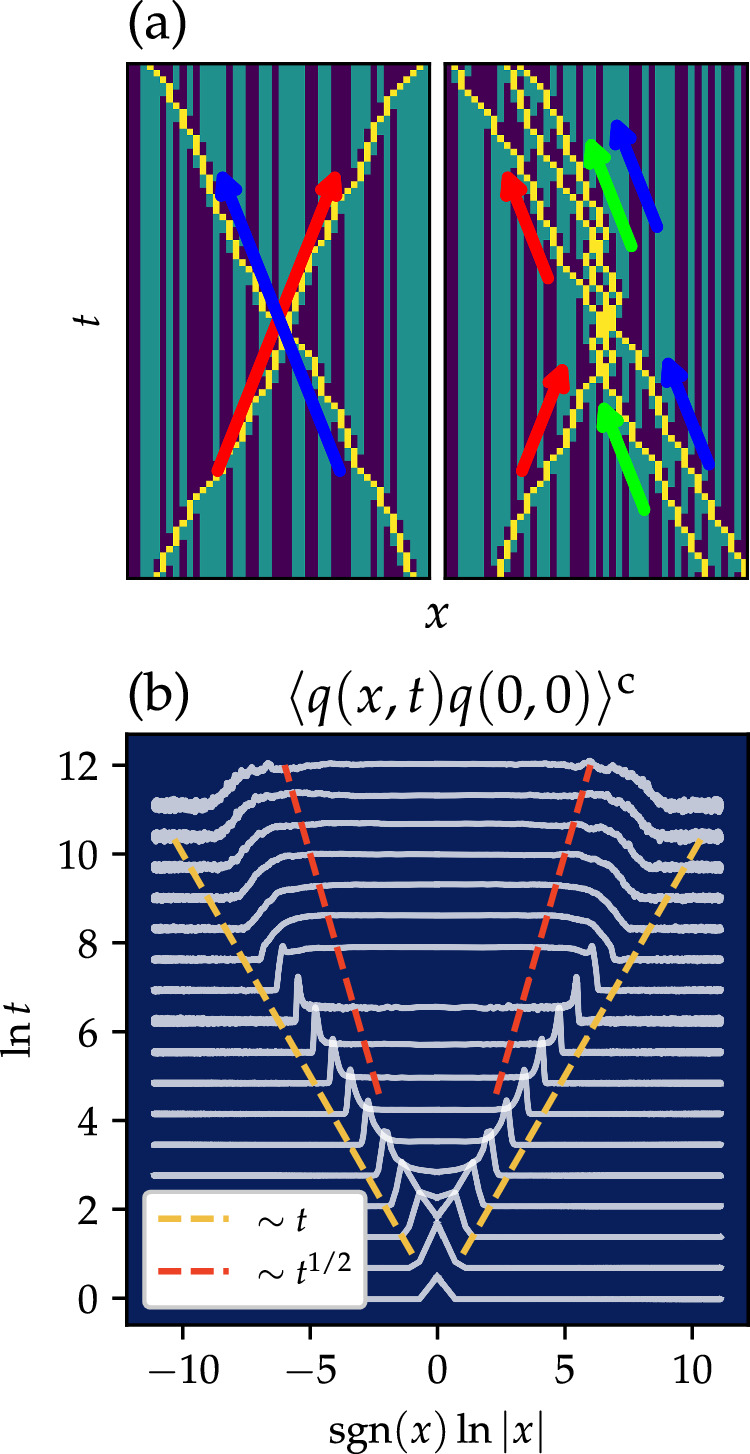
FPUT phenomenology in deterministic circuits, illustrated with gate (*d*, *σ*) = (3, 2312). **a** Trajectories of collisions between $\left|2\right\rangle$ s in a bath of $\left|0\right\rangle$ s and $\left|1\right\rangle$ s. For two-particle collisions, the $\left|2\right\rangle$ s conserve momentum (left). For three-or-more particle collisions, momentum is not conserved in general (right). **b** Due to approximate integrability at low fillings, the correlations (white lines) show ballistic ~*t* spreading at early times (dashed yellow lines). Eventually, the three-or-more particle collisions facilitate diffusive ~*t*^1/2^ correlations (dashed red lines). Here, *β* = −1.5, and simulations were carried out with *L* = 2^16^ and *T* = 2^14^ and averaged over 10^4^ samples. Source data are provided as a Source Data file.

Indeed, looking the trajectories of $\left|2\right\rangle$ s, at two-body collisions, they pass through each other (Supplementary Sec. F), as left of Fig. 6a. This is similar to what happens by energy and momentum conservation in Hamiltonian particle systems. Only when three or more $\left|2\right\rangle$ s meet is there a non-trivial interaction that breaks their linear trajectories, as right of Fig. 6a. This facilitates the eventual diffusion. Therefore, at low density of $\left|2\right\rangle$’s and short timescales, all trajectories are ballistic as in integrable systems, only becoming diffusive at later times, see Fig. 6b.

• **Anomalous current fluctuations**. Shocks and KPZ superdiffusion are observed in generic hydrodynamic equations. However, in certain systems satisfying linear degeneracy conditions, shock formation is absent^26^. Intuitively speaking, linear degeneracy means that hydrodynamic modes do not self-interact. This includes models with vanishing currents, such as circuit (*d*, *σ*) = (3, 2312) discussed previously, but also integrable systems. In linear degenerate systems, corrections to Euler hydrodynamics are typically expected to be diffusive^7^. However, more nuanced universal behavior can emerge. Recently, there has been interest in anomalous diffusion due to anomalous current fluctuations. This behavior has also been observed classical brickwork circuits in refs. ^45, 46^, which correspond to (*d*, *σ*) = (3, 12990) in our notation.

## Discussion

In this paper we introduced powerful but straightforward methods to deduce the conserved quantities of classical deterministic brickwork circuits and their exact hydrodynamic equations. Harnessing the efficiency of performing microscopic simulations of such circuits, we convincingly verified the hydrodynamic predictions. As an important example, we described the behaviour of circuit (*d*, *σ*) = (3, 996), to our knowledge the first case of a deterministic, closed system with a single conserved quantity whose microscopics yield the Burgers equation and KPZ superdiffusion correlations at large scales. We stress the simplicity and generality of our setting here, compared to the complications in the very few other deterministic interacting models (integrable systems and certain classical 1D chains) where such formulations are possible^3^. Because of this, the circuit approach should help understand all aspects of hydrodynamic theories and the way they emerge from microscopics.

The techniques we developed readily extend to higher-dimensional and to quantum circuits. In the quantum case, the continuum of possible two-body gates would allow to mimic the behavior of Hamiltonian systems with a smoothly-varying external potential. Hydrodynamic theories for quantum circuits are particularly interesting as they provide a way to benchmark the performance of quantum computers at large scale^47^. Since hydrodynamic evolution in *D* > 1 generically displays turbulence rather than shocks, an important avenue for research is to find simple circuit models that can provide insight into the poorly understood physics of turbulence^1^.

## Methods

### Labelling the gates

The label *σ* that defines a gate is defined as follows. We first identify each two-site configuration $(a,{a}^{{\prime} })$ in ${{\mathcal{D}}}\times {{\mathcal{D}}}$ with a number $A=da+{a}^{{\prime} }$, such that (0, 0) ↔ 0, (0, 1) ↔ 1, until (*d* − 1, *d* − 1) ↔ *d*^2^ − 1. Then the action of a gate corresponds to a permutation $(0,1,..,{d}^{2}-1)\to {A}_{0:{d}^{2}-1}=({A}_{0},..,{A}_{{d}^{2}-1})$. The set of all permutations $\{{A}_{0:{d}^{2}-1}\}$ can be then indexed in lexicographic order: *σ* = 0 corresponds to the trivial permutation ${A}_{0:{d}^{2}-1}=(0,1,2,..,{d}^{2}-3,{d}^{2}-2,{d}^{2}-1)$, *σ* = 1 to ${A}_{0:{d}^{2}-1}=(0,1,2,..,{d}^{2}-3,{d}^{2}-1,{d}^{2}-2)$, and so on, all the way to *σ* = (*d*^2^)! − 1 for ${A}_{0:{d}^{2}-1}=({d}^{2}-1,{d}^{2}-2,{d}^{2}-3,..,2,1,0)$.

### Finding the conserved quantities

Here, present an exact method to determine if there exists CQs with densities of *locality* up to (2*l* − 1) for some *l*, i.e., supported on (2*l* − 1) sites. We note that there is an existing method to find CQs^48,49^. However, our method is specifically designed for the circuit setup and furthermore also allows us to study CQs with non-trivial periodicities, which are shown to contribute to thermalisation, see Fig. 2b, e, f.

We define the right translation matrix 29which shifts configurations $\cdot \cdot {\left|a\right\rangle }_{-1}{\left|b\right\rangle }_{0}{\left|c\right\rangle }_{1}\cdot \cdot \to \cdot \cdot {\left|a\right\rangle }_{0}{\left|b\right\rangle }_{1}{\left|c\right\rangle }_{2}\cdot \cdot$. Evolution over two steps can then be written 30Consider some $\left\langle F\right|$ that satisfies $\langle F|{{{{\mathbf{T}}}}}^{2}=\langle F|{\mu }^{2}$ and $\langle F|{{{\mathbf{U}}}}=\langle F|{\lambda }^{2}$ (note $[{{{{\mathbf{T}}}}}^{2},{{{\mathbf{U}}}}]=0$). The latter condition can be written $\langle F|{({{{\bf{u}}}}_{1}{{{\mathbf{T}}}})}^{2}{{{{\mathbf{T}}}}}^{-2}=\langle F|{\lambda }^{2}$, and since $[{{{\bf{u}}}}_{1}{{{\mathbf{T}}}},{{{{\mathbf{T}}}}}^{2}]=0$, this means that $\langle F|{{{\bf{u}}}}_{1}{{{\mathbf{T}}}}=\langle F|\lambda \mu$. As both **u**_1_ and ${{{\mathbf{T}}}}$ are permutations, *μ* and *λ* must be phases: if they are roots of unity of order *m* and *n*, respectively, then ⟨F∣ is a CQ with spatial periodicity *m* and temporal periodicity *n*, i.e., it is conserved after *n* iterations of time evolution.

Now consider an ansatz for the CQ,

31 $$\left\langle F\right|={\sum }_{j\,{{\rm{odd}}}}{\mu }\,^{j}\left(\cdot \cdot \left\langle -\right|{\left\langle f\right|}_{j:j+2l-1}\left\langle -\right|\cdot \cdot \right) = :{\sum }_{j{{\rm{odd}}}}{\mu }^{j}\left\langle {F}_{j}\right|$$

with $\langle F|{{{{\mathbf{T}}}}}^{2}=\langle F|{\mu }^{2}$ by definition. Without loss of generality, we restrict to CQs of the type

32 $${\left\langle f\right|}_{1:2l}={\left\langle \,{f}_{{{\rm{o}}}}\right|}_{1:2l-1}{\left\langle -\right|}_{2l}+{\left\langle -\right|}_{1}{\left\langle \,{f}_{{{\rm{e}}}}\right|}_{2:2l},$$

corresponding to (2*l* − 1)-local CQs that are different starting on even and odd sites, and write the CQ as

33 $$\left\langle F\right|={\sum }_{i\,{{\rm{odd}}}}{\mu }^{i}\left(\left\langle {F}_{i}^{{{\rm{o}}}}\right|+\left\langle {F}_{i+1}^{{{\rm{e}}}}\right|\right),$$

where $\left\langle {F}_{i}^{{{\rm{o}}},{{\rm{e}}}}\right|=\left(\cdot \cdot \left\langle -\right|{\left\langle \,{f}_{{{\rm{o}}},{{\rm{e}}}}\right|}_{i:i+2l-1}\left\langle -\right|\cdot \cdot \right)$. Considering the action of **u**_1_ on $\left\langle F\right|$, we find that any $\left\langle \,{f}_{{{\rm{o}}}}\right|$, $\left\langle \,{f}_{{{\rm{e}}}}\right|$ that satisfies

34 $$\left(\left\langle \,{f}_{{{\rm{o}}}}\right|\left\langle -\right|+\left\langle -\right|\left\langle \,{f}_{{{\rm{e}}}}\right|\right){{\mathsf{u}}}_{1:2l}=\left({\mu }^{-1}\left\langle \,{f}_{{{\rm{e}}}}\right|\left\langle -\right|+\mu \left\langle -\right|\left\langle \,{f}_{{{\rm{o}}}}\right|\right)\lambda,$$

where ${{\mathsf{u}}}_{1:2l}=\underset{i=1,{{\rm{odd}}}}{\overset{2l-1}{\bigotimes }}{{\mathsf{u}}}_{i:i+1}$. Eq. (34) is a linear equation for $\left\langle \,{f}_{{{\rm{o}}}}\right|\oplus \left\langle \,{f}_{{{\rm{e}}}}\right|$ that can be solved to determine if there are any CQs of the form Eq. (33). For each gate ${\mathsf{u}}$, we solve Eq. (34) to determine if there are any CQs for given (*l*, *μ*, *λ*), scanning roots of unity of order (*m*, *n*) for (*μ*, *λ*), starting from *m* = *n* = 1 and increasing them systematically. Further details, such as gauge fixing, are given in Supplementary Sec. B.

### Deducing the manifold of Gibbs states

To detect if there is zero, one, or more CQs, we proceed as follows: (i) we choose two random quantities *A*_*l*_, *B*_*l*_, supported on *l* sites and orthogonal to the flat state and each other (i.e., $\left\langle {A}_{l}| {B}_{l}\right\rangle=0$); (ii) we construct an initial many-body probability distribution with spatial periodicity *m* as the product of a random *m*-body probability *p*(*a*_1:*m*_); (iii) we sample an initial configuration from this initial distribution; (iv) we then run the dynamics for *L* ≫ *T* ≫ *t*_thermo_ such that boundary conditions are not seen but the system relaxes.

We obtain *t*_thermo_ by plotting observables with respect to time and locating when they plateau. We estimate the thermal averages ${\left\langle {A}_{l}\right\rangle }_{\vec{\beta }}$ and ${\left\langle {B}_{l}\right\rangle }_{\vec{\beta }}$ as running averages over *n* (alternate) timesteps after thermalisation. By parametrically ploting two such numerical estimates over a range of initial states we can infer *N*_cq_. If there are no CQs, the plot should converge to a single point corresponding to the single maximum entropy state. If *N*_cq_ = 1, the plot will converge to a one-dimensional curve on the plane, while for *N*_cq_ > 1, it will become two-dimensional. In Fig. 2a we show such plot for the circuit (*d*, *σ*) = (3 996), for *l* = 4, *m* = 2, 4 and *n* = 2. We see that the numerical averages converge on a one-dimensional manifold indicating only a single CQ. The numerics agrees well the theory, confirming that (3 996) is a SG.

The generalisation to *N*_cq_ > 1 is straightforward. For example, if two CQs are expected, then expectations of three randomly chosen observables should occupy a two-dimensional embedded. We show such case in Fig. 2b for circuit (*d*, *σ*) = (3, 229117). In higher dimensions, where visualisation becomes difficult, box-counting dimensions^50^ could be used to determine the dimension of the sub-manifold and thus confirm *N*_cq_.

### Microscopic continuity equations

After the CQs are verified, we construct the microscopic continuity equations^3,34^. For the *N*_cq_ CQs, with spatial periodicities ${\{{m}_{\alpha }\}}_{1:{N}_{{{\rm{cq}}}}}$, we define a supercell of size $m={{\rm{lcm}}}({\{{m}_{\alpha }\}}_{1:{N}_{{{\rm{cq}}}}},2)$, and charge in the supercell

35 $$\left\langle {q}_{i}^{(\alpha )}\right|=\frac{1}{m}{\sum }_{j{{\rm{even}}}}^{m-2}{\mu }_{\alpha }^{j}\left\langle {F}_{i+j}^{(\alpha )}\right|\,.$$

Then, given temporal periodicities ${\{{n}_{\alpha }\}}_{1:{N}_{{{\rm{cq}}}}}$, we consider the change in charge in this supercell after a superunit of time $n={{\rm{lcm}}}({\{{n}_{\alpha }\}}_{1:{N}_{{{\rm{cq}}}}},2)$. This leads to the microscopic continuity equation

36 $$\frac{1}{n}\left(\left\langle {q}_{i,n}^{(\alpha )}\right|-\left\langle {q}_{i,0}^{(\alpha )}\right|\right)=-\frac{1}{m}\left(\left\langle {{{\rm{J}}}}_{i+m,0}^{(\alpha )}\right|-\left\langle {{{\rm{J}}}}_{i,0}^{(\alpha )}\right|\right).$$

Here, $\langle {q}_{i,\tau }^{(\alpha )}|=\langle {q}_{i}^{(\alpha )}|{{{{\mathbf{U}}}}}^{\tau /2}$ is the time-evolved charge, $\left\langle {{{\rm{J}}}}_{i}^{(\alpha )}\right|$ is the current, and $\langle {{{\rm{J}}}}_{i+m,0}^{(\alpha )}|=\langle {{{\rm{J}}}}_{i,0}^{(\alpha )}|{{{{\mathbf{T}}}}}^{-m}$ is the current shifted by *m* sites. For a SCQ 37The general case is detailed in Supplementary Sec. C.

### Implementation of Rankine–Hugoniot conditions

After the onset of the shock, the configuration is no longer differentiable in *x*. Therefore it cannot be a solution to

38 $${\partial }_{t}q+{\partial }_{x}{{\rm{J}}}(q)=0.$$

Instead, it is only a “weak” solution, i.e., a solution that satisfies the integrated version of Eq. (38), i.e., for all *x*_1_ < *x*_2_, *t*_1_ < *t*_2_ we have^26^:

39 $${\int }_{{x}_{1}}^{{x}_{2}} {{\rm{d}}}xq(x,{t}_{2})-{\int }_{{x}_{1}}^{{x}_{2}}{{\rm{d}}}xq(x,{t}_{2})+{\int }_{{t}_{1}}^{{t}_{2}}{{\rm{d}}}t{{\rm{J}}}(q({x}_{2},t))-{\int }_{{t}_{1}}^{{t}_{2}}{{\rm{d}}}t{{\rm{J}}}(q({x}_{1},t))=0.$$

For differentiable solutions, both conditions are equivalent. However, for discontinuous solutions, the derivative cannot be defined and thus Eq. (38) is ill-posed. Nevertheless, integration over discontinuous solutions is still well-defined, making Eq. (39) a suitable formulation. Unfortunately, while differentiable solutions (called strong solutions) are unique, weak solutions are formally not^26^. For instance, while on physical grounds, we expect shocks to increase entropy, there also exist solutions that always decrease entropy. Therefore, we require further assumptions to fix a solution.

The Rankine–Hugoniot conditions are guided by charge conservation. Formally, entering a discontinuous solution

40 $$q(x,t)=\Delta q\theta (x-{x}_{{{\rm{shock}}}}(t))+{q}_{{{\rm{smooth}}}}(x,t)$$

into Eq. (38), we obtain

41 $$\Delta q\delta (x-{x}_{{{\rm{shock}}}}(t)) \frac{{{\rm{d}}}}{{{\rm{d}}}t}{x}_{{{\rm{shock}}}}(t)+\Delta {{\rm{J}}}\delta (x-{x}_{{{\rm{shock}}}}(t))+\cdots=0,$$

where the dots represent the smooth part of the solution. From Eq. (38), we can read off

42 $${v}_{{{\rm{shock}}}}=\frac{{{\rm{d}}}}{{{\rm{d}}}t}{x}_{{{\rm{shock}}}}(t)=\frac{\Delta {{\rm{J}}}}{\Delta q}.$$

This is said to be the velocity of a shock only if Δ*v* < 0, i.e., if the velocity behind the shock is larger than the velocity in front of the shock. The last assumption breaks time-reversal symmetry and is responsible for the entropy increase of a shock (if we instead required that Δ*v* > 0, shocks would always decrease entropy).

This can be implemented numerically using the following well-known “upwind” scheme,

43 $$	\frac{q({t}_{j}+\Delta t,{x}_{i})-q({t}_{j},{x}_{i})}{\Delta t}\\=	 -\frac{1}{\Delta x}\left\{\begin{array}{ll}{{\rm{J}}}(q({t}_{j},{x}_{j}))-{{\rm{J}}}(q({t}_{j},{x}_{j}-\Delta x)) & v(q({t}_{j},{x}_{i})) > 0\\ {{\rm{J}}}(q({t}_{j},{x}_{j}+\Delta x))-{{\rm{J}}}(q({t}_{j},{x}_{j})) & v(q({t}_{j},{x}_{i})) < 0.\end{array}\right.$$

Here *x*_*i*_, *t*_*j*_ are the space and time-discretisations (whose spacings are equidistant). This scheme does not explicitly implement the Rankine–Hugoniot conditions, but does so implicitly. The scheme explicitly conserves the total charge ∑_*i*_ *q*(*t*_*j*_, *x*_*i*_) = ∑_*i*_ *q*(*t*_0_, *x*_*i*_), hence it has Eq. (42) “built in”. Note that this scheme explicitly breaks time-reversal symmetry, since the discretization of the spatial derivative on the RHS of Eq. (43) depends on the sign of the velocity.

## Supplementary information

Supplementary Information

Transparent Peer Review file

## Source data

Source Data

## Data Availability

All figures can be reproduced from the equations provided in the main text, methods, and supplementary information. Source data for figures are provided with this paper. Source data are provided with this paper.

## Code availability

The algorithms supporting this work are fully described by the main text, methods, and supplementary information.
