# Supplementary material for: Circuits as a simple platform for the emergence of hydrodynamics in deterministic chaotic many-body systems
Source: Nat Commun. 2026 Jun 30;17:8152. doi: 10.1038/s41467-026-74457-3 (PMC13458585; doi:10.1038/s41467-026-74457-3)
Supplement: Supplementary file 1 [file 41467_2026_74457_MOESM1_ESM.pdf]

Supplementary Information for "Circuits as a simple platform for the emergence of hydrodynamics in deterministic chaotic many-body systems"

Sun Woo P. Kim,[1,*] Friedrich Hübner,[2] Juan P. Garrahan,[3,4] and Benjamin Doyon[2]

[1]*Department of Physics, King's College London, Strand, London WC2R 2LS, United Kingdom*
[2]*Department of Mathematics, King's College London, Strand, London WC2R 2LS, United Kingdom*
[3]*School of Physics and Astronomy, University of Nottingham, Nottingham NG7 2RD, United Kingdom*
[4]*Centre for the Mathematics and Theoretical Physics of Quantum Non-Equilibrium Systems, University of Nottingham, Nottingham NG7 2RD, United Kingdom*

A. GATES FOR $d = 2$

σ	Name	Rules	# CQs with support $\leq l$										(Likely) classification
			Normal					Staggered					
			1	2	3	4	5	1	2	3	4	5	
0	Identity	$00 \to 00, 01 \to 01, 10 \to 10, 11 \to 11$	3	9	33	129	513	13	49	193	769	3073	Integrable
1	West	$00 \to 00, 01 \to 01, 10 \to 11, 11 \to 10$	1	1	1	1	1	1	1	1	1	1	Fully chaotic
2	SWAP	$00 \to 00, 01 \to 10, 10 \to 01, 11 \to 11$	3	5	9	17	33	13	25	49	97	193	Integrable
3		$00 \to 00, 01 \to 10, 10 \to 11, 11 \to 01$	2	4	7	13	25	7	19	37	73	145	Integrable
4		$00 \to 00, 01 \to 11, 10 \to 01, 11 \to 10$	2	4	7	13	25	7	19	37	73	145	Integrable
5	East	$00 \to 00, 01 \to 11, 10 \to 10, 11 \to 01$	1	1	1	1	1	1	1	1	1	1	Fully chaotic
6	Inverted West	$00 \to 01, 01 \to 00, 10 \to 10, 11 \to 11$	1	1	1	1	1	1	1	1	1	1	Fully chaotic
7		$00 \to 01, 01 \to 00, 10 \to 11, 11 \to 10$	1	5	17	65	257	13	49	193	769	3073	Integrable
8		$00 \to 01, 01 \to 10, 10 \to 00, 11 \to 11$	2	4	7	13	25	7	19	37	73	145	Integrable
9		$00 \to 01, 01 \to 10, 10 \to 11, 11 \to 00$	1	1	1	1	1	1	1	1	1	1	Fully chaotic
10		$00 \to 01, 01 \to 11, 10 \to 00, 11 \to 10$	3	5	9	17	33	13	25	49	97	193	Integrable
11		$00 \to 01, 01 \to 11, 10 \to 10, 11 \to 00$	2	4	7	13	25	7	19	37	73	145	Integrable
12		$00 \to 10, 01 \to 00, 10 \to 01, 11 \to 11$	2	4	7	13	25	7	19	37	73	145	Integrable
13		$00 \to 10, 01 \to 00, 10 \to 11, 11 \to 01$	3	5	9	17	33	13	25	49	97	193	Integrable
14	Inverted East	$00 \to 10, 01 \to 01, 10 \to 00, 11 \to 11$	1	1	1	1	1	1	1	1	1	1	Fully chaotic
15		$00 \to 10, 01 \to 01, 10 \to 11, 11 \to 00$	2	4	7	13	25	7	19	37	73	145	Integrable
16		$00 \to 10, 01 \to 11, 10 \to 00, 11 \to 01$	1	5	17	65	257	13	49	193	769	3073	Integrable
17		$00 \to 10, 01 \to 11, 10 \to 01, 11 \to 00$	1	1	1	1	1	1	1	1	1	1	Fully chaotic
18		$00 \to 11, 01 \to 00, 10 \to 01, 11 \to 10$	1	1	1	1	1	1	1	1	1	1	Fully chaotic
19		$00 \to 11, 01 \to 00, 10 \to 10, 11 \to 01$	2	4	7	13	25	7	19	37	73	145	Integrable
20		$00 \to 11, 01 \to 01, 10 \to 00, 11 \to 10$	2	4	7	13	25	7	19	37	73	145	Integrable
21		$00 \to 11, 01 \to 01, 10 \to 10, 11 \to 00$	3	5	9	17	33	13	25	49	97	193	Integrable
22		$00 \to 11, 01 \to 10, 10 \to 00, 11 \to 01$	1	1	1	1	1	1	1	1	1	1	Fully chaotic
23	NOT	$00 \to 11, 01 \to 10, 10 \to 01, 11 \to 00$	3	9	33	129	513	13	49	193	769	3073	Integrable

SUPPLEMENTARY TABLE I. Exhaustive list of likely classification of all classical deterministic gates with local dimension $d = 2$. *Normal* CQs corresponds to conserved quantities with $\mu = 1$ and $\lambda = \pm 1$. *Staggered* CQs corresponds to ones where μ, λ are higher order roots of unity. We ran a search for λ^n, μ up to 12th roots of unity, $m, n \leq 12$. For the likely integrable gates, it may that more CQs exist at higher orders. Thus, the number only represents a lower bound. Note that there is always one CQ, corresponding to the flat state $\langle -| \langle -|$.

* swk34@cantab.ac.uk

B. FINDING CONSERVED QUANTITIES AND GAUGE FIXING

The naive approach (Eq. (34)) will yield a myriad of solutions. This is because we have not considered gauge freedom. Note that due to the summation over space, for some solution $\langle f|$, $\langle f| + \mu^{-2}\langle\vartheta, -, -| - \langle -, -, \vartheta|$ for some gauge field $\langle\vartheta|$ of length $2(l-1)$, gives the same $\langle F|$. Here, we used a shorthand $\langle f, g| := \langle f|\,\langle g|$. Therefore any $\langle f|$ satisfying

$$\Big(\langle f_{\rm o}, -| + \langle -, f_{\rm e}|\Big)\mathsf{u}_{1:2l} = \Big(\mu^{-2}\langle f_{\rm e}, -| + \langle -, f_{\rm o}|\Big)\lambda\mu + \mu^{-2}\langle C, -, -| - \langle -, -, C|, \tag{B.1}$$

for any $\langle C|$ of length $2(l-1)$, results in a valid local CQ. There is one additional gauge freedom. For given any solution $\langle f_{\rm e}|, \langle f_{\rm o}|$, the gauge transformation

$$\langle f_{\rm o}| \to \langle f'_{\rm o}| = \langle f_{\rm o}| + \langle -, \chi|, \qquad \langle f_{\rm e}| \to \langle f'_{\rm e}| = \langle f_{\rm e}| - \langle \chi, -|, \tag{B.2}$$

for some gauge field $\langle\chi|$ of length $2(l-1)$, also gives the same $\langle F|$. The previous gauge freedom can be assigned to $\langle f_{\rm e}|$, $\langle f_{\rm o}|$, as

$$\langle f_{\rm o}| \to \langle f'_{\rm o}| = \langle f_{\rm o}| + \mu^{-2}\langle \vartheta, -|, \qquad \langle f_{\rm e}| \to \langle f'_{\rm e}| = \langle f_{\rm e}| - \langle -, \vartheta|. \tag{B.3}$$

We therefore have two gauge freedoms $\langle\vartheta|$ and $\langle\chi|$, each with $d^{2(l-1)}$ degrees of freedom, with a total of $d^{4(l-1)}$. Suppose that we have a solution obeying Eq. (B.1). We can first write $\langle f|$ in terms of $\langle f'|$ to cancel $\langle C|$. After this, any gauge that will not produce any $\langle C|$ term requires that $\langle\vartheta|\,\mathsf{u}_{1:2l-2} = -\lambda\mu\,\langle\chi|$. With the remaining $d^{2(l-1)}$ degrees of freedom, we set particular values of $\langle f|$ to zero for various cases. For example, for the case where $\mu \neq 1$, we set $\langle f|0, 0, a_3, .., a_{2l}\rangle = 0\;\forall(a_3, .., a_{2l}) \neq (0, .., 0)$, and for the case where $\mu = 1$, we set $\langle f_{\rm e}|0, \cdot\cdot, 0\rangle = 0$.

For a given gate σ, we use the null space method, where we set l, μ, and λ and look for the null space of the condition Eq. (34) subject to additional gauge-fixing constraints. We start from $l = m = n = 1$ case then increase the search space for higher roots of unity to determine conserved quantities of higher periodicity and locality.

C. CONSTRUCTING HYDRODYNAMIC EQUATIONS IN THE GENERAL CASE

Consider a species of conserved quantity labelled by α. Eq. (34) can also be written as

$$\Big(\langle F_i^{{\rm o},(\alpha)}| + \langle F_{i+1}^{{\rm e},(\alpha)}|\Big)\mathbf{u}_1 = \lambda_\alpha\mu_\alpha\left(\langle F_i^{{\rm o},(\alpha)}|\mathbf{T}^{-1} + \frac{1}{\mu_\alpha^2}\langle F_{i+1}^{{\rm e},(\alpha)}|\mathbf{T}\right), \tag{C.1}$$

where i is odd. Then for the time-evolved charge $\langle F_{i,\tau}^{(\alpha)}|$, where $\langle F_{i,0}^{(\alpha)}| = \langle F_i^{(\alpha)}| = \langle F_i^{{\rm o},(\alpha)}| + \langle F_{i+1}^{{\rm e},(\alpha)}|$, we have

$$\langle F_{i,2}^{(\alpha)}| - \lambda_\alpha^2\langle F_{i,0}^{(\alpha)}| = \langle F_i^{(\alpha)}|\mathbf{U} - \lambda_\alpha^2\langle F_i^{(\alpha)}| \tag{C.2}$$

$$= \langle F_i^{(\alpha)}|\mathbf{u}_1\mathbf{T}\mathbf{u}_1\mathbf{T}^{-1} - \lambda_\alpha^2\langle F_i^{(\alpha)}| \tag{C.3}$$

$$= \lambda_\alpha\mu_\alpha\left(\langle F_i^{{\rm o},(\alpha)}|\mathbf{T}^{-1} + \frac{1}{\mu_\alpha^2}\langle F_{i+1}^{{\rm e},(\alpha)}|\mathbf{T}\right)\mathbf{T}\mathbf{u}_1\mathbf{T}^{-1} - \lambda_\alpha^2\langle F_i^{(\alpha)}| \tag{C.4}$$

$$= \lambda_\alpha\mu_\alpha\left(\langle F_i^{{\rm o},(\alpha)}| + \frac{1}{\mu_\alpha^2}\Big(\langle F_i^{(\alpha)}| - \langle F_i^{{\rm o},(\alpha)}|\Big)\mathbf{T}^2\right)\mathbf{u}_1\mathbf{T}^{-1} - \lambda_\alpha^2\langle F_i^{(\alpha)}|. \tag{C.5}$$

At this point we use the fact that $\mathbf{T}^2\mathbf{u}_1\mathbf{T}^{-2} = \mathbf{u}_1$ to simplify $\mathbf{T}^2\mathbf{u}_1\mathbf{T}^{-1} = \mathbf{u}_1\mathbf{T}$, then use the identity Eq. (C.1) again on $\langle F_i^{(\alpha}|$ to get

$$\langle F_{i,2}^{(\alpha)}| - \lambda_\alpha^2\langle F_{i,0}^{(\alpha)}| = \lambda_\alpha\mu_\alpha\langle F_i^{{\rm o},(\alpha)}|\mathbf{u}_1\mathbf{T}\mathbf{T}^{-2} + \frac{\lambda_\alpha^2}{\mu_\alpha^2}\langle F_{i+1}^{{\rm e},(\alpha)}|\mathbf{T}^2 - \frac{\lambda_\alpha}{\mu_\alpha}\langle F_i^{{\rm o},(\alpha)}|\mathbf{u}_1\mathbf{T} - \lambda^2\langle F_{i+1}^{{\rm e},(\alpha)}|. \tag{C.6}$$

Now defining "bare" current as

$$\langle G_i^{(\alpha)}| := -\lambda_\alpha\mu_\alpha^{-1}\langle F_i^{{\rm o},(\alpha)}| + \lambda^2\mu^{-2}\langle F_{i+1}^{{\rm e},(\alpha)}|\mathbf{T}^{-2}, \tag{C.7}$$

we have

$$\langle F_{i,2}^{(\alpha)}| - \lambda_\alpha^2\langle F_{i,0}^{(\alpha)}| = -\Big(\mu_\alpha^2\langle G_{i+2,0}^{(\alpha)}| - \langle G_{i,0}^{(\alpha)}|\Big), \tag{C.8}$$

where $\langle G^{(\alpha)}_{i+2}| = \langle G^{(\alpha)}_{i}|\mathbf{T}^{-2}$.

Now we consider time evolution of charge defined in Eq. (35) by superunit of time n. Since by definition $\lambda_\alpha^{-n} = \lambda_\alpha^{-0} = 1$, we have

$$\langle q^{(\alpha)}_{i,n}| - \langle q^{(\alpha)}_{i,0}| = \lambda_\alpha^{-n}\langle q^{(\alpha)}_{i,n}| - \lambda_\alpha^{-0}\langle q^{(\alpha)}_{i,0}| \tag{C.9}$$

$$= \left(\lambda_\alpha^{-n}\langle q^{(\alpha)}_{i,n}| - \lambda_\alpha^{-(n-2)}\langle q^{(\alpha)}_{i,n-2}|\right) + \left(\lambda_\alpha^{-(n-2)}\langle q^{(\alpha)}_{i,n-2}| - \lambda_\alpha^{-(n-4)}\langle q^{(\alpha)}_{i,n-4}|\right) + \cdot\cdot + \left(\lambda_\alpha^{-2}\langle q^{(\alpha)}_{i,2}| - \lambda_\alpha^{-0}\langle q^{(\alpha)}_{i,0}|\right) \tag{C.10}$$

$$= \sum_{\tau=0,\text{ even}}^{n-2} \left(\lambda_\alpha^{-(\tau+2)}\langle q^{(\alpha)}_{i,\tau+2}| - \lambda_\alpha^{-\tau}\langle q^{(\alpha)}_{i,\tau}|\right). \tag{C.11}$$

Expanding $\langle q^{(\alpha)}_{i,\tau}|$,

$$\langle q^{(\alpha)}_{i,n}| - \langle q^{(\alpha)}_{i,0}| = \frac{1}{m}\sum_{\tau=0,\text{ even}}^{n-2}\sum_{j=0,\text{ even}}^{m-2} \left(\lambda_\alpha^{-(\tau+2)}\mu_\alpha^j\langle F^{(\alpha)}_{i+j,\tau+2}| - \lambda_\alpha^{-\tau}\mu_\alpha^j\langle F^{(\alpha)}_{i+j,\tau}|\right) \tag{C.12}$$

$$= \frac{1}{m}\sum_{\tau=0,\text{ even}}^{n-2}\sum_{j=0,\text{ even}}^{m-2} \lambda_\alpha^{-\tau}\mu_\alpha^j\left(\langle F^{(\alpha)}_{i+j,\tau+2}| - \lambda_\alpha^2\langle F^{(\alpha)}_{i+j,\tau}|\right) \tag{C.13}$$

$$= \frac{1}{m}\sum_{\tau=0,\text{ even}}^{n-2}\sum_{j=0,\text{ even}}^{m-2} \lambda_\alpha^{-\tau}\mu_\alpha^j\left(\langle F^{(\alpha)}_{i+j,2}| - \lambda_\alpha^2\langle F^{(\alpha)}_{i+j,0}|\right)\mathbf{U}^{\tau/2}. \tag{C.14}$$

Using the bare continuity relation Eq. (C.8), we have

$$\langle q^{(\alpha)}_{i,n}| - \langle q^{(\alpha)}_{i,0}| = -\frac{1}{m}\sum_{\tau=0,\text{ even}}^{n-2}\sum_{j=0,\text{ even}}^{m-2} \lambda_\alpha^{-\tau}\mu_\alpha^j\left(\mu_\alpha^2\langle G^{(\alpha)}_{i+j+2,0}| - \langle G^{(\alpha)}_{i+j,0}|\right)\mathbf{U}^{\tau/2} \tag{C.15}$$

$$= -\frac{1}{m}\sum_{\tau=0,\text{ even}}^{n-2}\sum_{j=0,\text{ even}}^{m-2} \lambda_\alpha^{-\tau}\mu_\alpha^j\left(\mu_\alpha^2\langle G^{(\alpha)}_{i+j+2,0}| - \langle G^{(\alpha)}_{i+j,0}|\right)\mathbf{U}^{\tau/2} \tag{C.16}$$

$$= -\frac{1}{m}\sum_{\tau=0,\text{ even}}^{n-2}\sum_{j=0,\text{ even}}^{m-2} \lambda_\alpha^{-\tau}\mu_\alpha^j\left(\mu_\alpha^2\langle G^{(\alpha)}_{i+j+2,0}| - \langle G^{(\alpha)}_{i+j,0}|\right)\mathbf{U}^{\tau/2} \tag{C.17}$$

$$= -\frac{1}{m}\left(\sum_{\tau=0,\text{ even}}^{n-2} \lambda_\alpha^{-\tau}\left(\mu_\alpha^m\langle G^{(\alpha)}_{i+m,0}| - \mu_\alpha^0\langle G^{(\alpha)}_{i,0}|\right)\mathbf{U}^{\tau/2}\right) \tag{C.18}$$

$$= -\frac{1}{m}\left(\sum_{\tau=0,\text{ even}}^{n-2} \lambda_\alpha^{-\tau}\left(\langle G^{(\alpha)}_{i+m,0}| - \langle G^{(\alpha)}_{i,0}|\right)\mathbf{U}^{\tau/2}\right). \tag{C.19}$$

Then, defining current as

$$\langle \mathrm{J}^{(\alpha)}_i| = \frac{1}{n}\sum_{\tau=0,\text{ even}}^{n-2}\lambda_\alpha^{-\tau}\langle G^{(\alpha)}_{i,0}|\mathbf{U}^{\tau/2}, \tag{C.20}$$

we finally obtain the microscopy continuity Eq. (36) of the main text.

Next, we calculate the expectation on thermal states $|p_{\vec{\beta}}\rangle$. Note the two properties,

$$\mathbf{T}^2|p_{\vec{\beta}}\rangle = \frac{1}{Z(\vec{\beta})}\sum_{\boldsymbol{a}}\exp\left[-\sum_\alpha \beta_\alpha\langle F^{(\alpha)}|\boldsymbol{a}\rangle\right]\mathbf{T}^2|\boldsymbol{a}\rangle = \frac{1}{Z(\vec{\beta})}\sum_{\boldsymbol{a}}\exp\left[-\sum_\alpha \beta_\alpha\langle F^{(\alpha)}|\mathbf{T}^{-2}|\boldsymbol{a}\rangle\right]|\boldsymbol{a}\rangle \tag{C.21}$$

$$= \frac{1}{Z(\vec{\beta})}\sum_{\boldsymbol{a}}\exp\left[-\sum_\alpha \frac{\beta_\alpha}{\mu_\alpha^2}\langle F^{(\alpha)}|\boldsymbol{a}\rangle\right]|\boldsymbol{a}\rangle = |p_{\overrightarrow{(\beta/\mu^2)}}\rangle. \tag{C.22}$$

$$\mathbf{u}_1\mathbf{T}|p_{\vec{\beta}}\rangle = \frac{1}{Z(\vec{\beta})}\sum_{\boldsymbol{a}}\exp\left[-\sum_{\alpha}\beta_\alpha\langle F^{(\alpha)}|\mathbf{T}^{-1}\mathbf{u}_1^{-1}|\boldsymbol{a}\rangle\right]|\boldsymbol{a}\rangle = \frac{1}{Z(\vec{\beta})}\sum_{\boldsymbol{a}}\exp\left[-\sum_{\alpha}\frac{\beta_\alpha}{\mu_\alpha\lambda_\alpha}\langle F^{(\alpha)}|\boldsymbol{a}\rangle\right]|\boldsymbol{a}\rangle \tag{C.23}$$

$$= |p_{\overrightarrow{(\beta/\lambda\mu)}}\rangle. \tag{C.24}$$

Then the expectation value of the charge on the thermal state is

$$\langle q_i^{(\alpha)}\rangle_{\vec{\beta}} = \frac{1}{m}\sum_{j=0,\text{ even}}^{m-2}\mu_\alpha^j\langle F_{i+j}^{(\alpha)}\rangle_{\vec{\beta}} = \frac{1}{m}\sum_{j=0,\text{ even}}^{m-2}\mu_\alpha^j\langle F_i^{(\alpha)}\rangle_{\overrightarrow{(\beta\mu^j)}}. \tag{C.25}$$

The expectation of time-evolved bare current on thermal states is

$$\langle G_i^{(\alpha)}|\mathbf{U}^{\tau/2}|p_{\vec{\beta}}\rangle = \langle G_i^{(\alpha)}|(\mathbf{u}_1\mathbf{T}\mathbf{u}_1\mathbf{T}^{-1})^{\tau/2}|p_{\vec{\beta}}\rangle = \langle G_i^{(\alpha)}|p_{\overrightarrow{(\beta/\lambda^\tau)}}\rangle \tag{C.26}$$

$$= -\frac{\lambda_\alpha}{\mu_\alpha}\langle f_{\rm o}^{(\alpha)}\rangle_{\overrightarrow{(\beta/\lambda^{\tau+1}\mu)}} + \frac{\lambda_\alpha^2}{\mu_\alpha^2}\langle f_{\rm e}^{(\alpha)}\rangle_{\overrightarrow{(\beta/\lambda^{\tau}\mu^2)}} \tag{C.27}$$

Therefore the expectation of the current is

$$\langle {\rm j}_i^{(\alpha)}\rangle_{\vec{\beta}} = \frac{1}{n}\sum_{\tau=0,\text{ even}}^{n-2}\lambda_\alpha^{-\tau}\langle G_{i,0}^{(\alpha)}|\mathbf{U}^{\tau/2}|p_{\vec{\beta}}\rangle \tag{C.28}$$

$$= \frac{1}{n}\sum_{\tau=0,\text{ even}}^{n-2}\left(-\frac{1}{\lambda_\alpha^{\tau-1}\mu_\alpha}\langle f_{\rm o}^{(\alpha)}\rangle_{\overrightarrow{(\beta/\lambda^{\tau+1}\mu)}} + \frac{1}{\lambda_\alpha^{\tau-2}\mu_\alpha^2}\langle f_{\rm e}^{(\alpha)}\rangle_{\overrightarrow{(\beta/\lambda^{\tau}\mu^2)}}\right). \tag{C.29}$$

Finally Eq. (10) of main text can be resolved as

$$\partial_t\vec{q} = -\vec{\vec{B}}\vec{\vec{C}}^{-1}\partial_x\vec{q}, \tag{C.30}$$

where $[\vec{\vec{B}}]_{\alpha,\alpha'} = \partial{\rm j}^{(\alpha)}/\partial\beta_{\alpha'}$ and $[\vec{\vec{C}}]_{\alpha,\alpha'} = \partial q^{(\alpha)}/\partial\beta_{\alpha'}$.

D. DECOUPLING OF STAGGERED CQS FROM SIMPLE CQS

Now, we would like to consider a special case where conserved quantities are either non-staggered both in space and time, i.e. $\lambda_\alpha^2 = \mu_\alpha^2 = 1$, or staggered in space and time $\lambda_{\bar\alpha}^2 \neq 1, \mu_{\bar\alpha}^2 \neq 1$. For sake of slim notation, we will label by α a conserved quantity of the first type and by $\bar\alpha$ a conserved quantity of the second type. We want to show that in this case, whenever $\beta_{\bar\alpha} = 0$ for all $\bar\alpha$ the hydrodynamic equations for the non-staggered conserved quantities decouple from the staggered ones.

First, note that in this case we have

$$\overrightarrow{(\beta\lambda^\tau\mu^j)} = \vec{\beta}, \tag{D.1}$$

which inserted into (C.25) immediately gives

$$\langle q_i^{(\bar\alpha)}\rangle_{\vec{\beta}} = \frac{1}{m}\sum_{j=0,\text{ even}}^{m-2}\mu_{\bar\alpha}^j\langle F_i^{(\bar\alpha)}\rangle_{\vec{\beta}} = 0. \tag{D.2}$$

This cancellation happens since $\mu_{\bar\alpha}$ is an $m_{\bar\alpha}$ root of unity. Therefore, $\beta_{\bar\alpha} = 0$ corresponds to $\langle q_i^{(\bar\alpha)}\rangle_{\vec{\beta}} = 0$.

Similarly, we can compute the two matrices $\vec{\vec{C}}$ and $\vec{\vec{B}}$ and find that they are block-diagonal in $\alpha, \bar\alpha$:

$$[\vec{\vec{C}}]_{\alpha,\bar\alpha} = \frac{\rm d}{{\rm d}\beta_{\bar\alpha}}\langle q_i^{(\alpha)}\rangle_{\vec{\beta}}\bigg|_{\beta_{\bar\alpha}=0} = \frac{1}{m}\sum_{j=0,\text{ even}}^{m-2}\mu_{\bar\alpha}^j\frac{\rm d}{{\rm d}\beta_{\bar\alpha}}\langle F_i^{(\alpha)}\rangle_{\vec{\beta}}\bigg|_{\beta_{\bar\alpha}=0} = 0 \tag{D.3}$$

$$[\vec{\vec{B}}]_{\alpha,\bar\alpha} = \frac{\rm d}{{\rm d}\beta_{\bar\alpha}}\langle {\rm j}_i^{(\alpha)}\rangle_{\vec{\beta}}\bigg|_{\beta_{\bar\alpha}=0} = \frac{1}{n}\sum_{\tau=0,\text{ even}}^{n-2}\left(-\frac{1}{\lambda_{\bar\alpha}^{\tau-1}\mu_{\bar\alpha}}\frac{\rm d}{{\rm d}\beta_{\bar\alpha}}\langle f_{\rm o}^{(\alpha)}\rangle_{\vec{\beta}}\bigg|_{\beta_{\bar\alpha}=0} + \frac{1}{\lambda_{\bar\alpha}^{\tau-2}\mu_{\bar\alpha}^2}\frac{\rm d}{{\rm d}\beta_{\bar\alpha}}\langle f_{\rm e}^{(\alpha)}\rangle_{\vec{\beta}}\bigg|_{\beta_{\bar\alpha}=0}\right) = 0, \tag{D.4}$$

Since these two matrices are block-diagonal, also $\vec{A} = \vec{B}\vec{C}^{-1}$ is block-diagonal. Therefore the hydrodynamic equations read:

$$\partial_t q^{(\alpha)} + \sum_{\alpha'} [\vec{A}]_{\alpha,\alpha'} \partial_x q^{(\alpha')} = 0 \qquad \partial_t q^{(\bar{\alpha})} + \sum_{\bar{\alpha}'} [\vec{A}]_{\bar{\alpha},\bar{\alpha}'} \partial_x q^{(\bar{\alpha}')} = 0. \tag{D.5}$$

Recall that initially $q^{(\bar{\alpha})}(t=0,x) = 0$ and therefore the second equation is trivially solved by $q^{(\bar{\alpha})}(t,x) = 0$ for all times. Thus, our assumption of $\beta_{\bar{\alpha}} = 0$ remains valid throughout the hydrodynamic evolution and therefore the hydrodynamic equations decouple at all times.

We would like to stress that this decoupling only applies when $\beta_{\bar{\alpha}} = 0$, but not for generic values. In fact, computing higher order derivatives of $\langle q_i^{(\alpha)} \rangle_{\vec{\beta}}$ and $\langle \mathrm{J}_i^{(\alpha)} \rangle_{\vec{\beta}}$ at $\beta_{\bar{\alpha}} = 0$ one can see that they will eventually couple between α and $\bar{\alpha}$. For instance if $\lambda_{\bar{\alpha}} = \mu_{\bar{\alpha}} = i$, both $\frac{\mathrm{d}^2}{\mathrm{d}\beta_{\bar{\alpha}}^2} \langle q_i^{(\alpha)} \rangle_{\vec{\beta}} \big|_{\beta_{\bar{\alpha}}=0} \neq 0$ and $\frac{\mathrm{d}^2}{\mathrm{d}\beta_{\bar{\alpha}}^2} \langle \mathrm{J}_i^{(\alpha)} \rangle_{\vec{\beta}} \big|_{\beta_{\bar{\alpha}}=0} \neq 0$, signaling a coupling between both sets of conserved quantities.

E. DETAILS ON KARDAR-PARISI-ZHANG SCALING OF CORRELATIONS

Here we give more details on how we compare the correlation functions to the non-linear fluctuating hydrodynamic theory of [1] to predict not only KPZ superdiffusive scaling, but also the superdiffusion constant λ_{B}.

Our starting point is Eq. (21). Defining $\phi(x,t) = \delta q(x,t)/\sqrt{C}$, where

$$C = \int \mathrm{d}x \, \langle \delta q(x,0) \delta q(0,0) \rangle_\beta^{\mathrm{c}} \tag{E.1}$$

and neglecting higher order terms we find

$$\partial_t \phi = -\partial_x \left(v(q_0)\phi + \frac{1}{2} \mathrm{J}''(q_0) \sqrt{C} \phi^2 \right) \tag{E.2}$$

with initial fluctuations satisfying $\langle \phi(x,0) \rangle_\beta = 0$ and $\langle \phi(x,0)\phi(y,0) \rangle_\beta^{\mathrm{c}} = \delta(x-y)$.

According to non-linear fluctuating hydrodynamics [1] this equation has to be expanded to include *emergent* diffusion and noise

$$\partial_t \phi(x,t) = -\partial_x \left(v(q_0)\phi(x,t) + \frac{1}{2} \mathrm{J}''(q_0) \sqrt{C} \phi(x,t)^2 - D\partial_x \phi(x,t) + \sqrt{2D}\xi(x,t) \right). \tag{E.3}$$

Here D is the (undetermined) diffusion constant and $\xi(x,t)$ is white noise, i.e. a stochastic field with $\langle \xi(x,t) \rangle = 0$ and $\langle \xi(x,t)\xi(y,s) \rangle = \delta(x-y)\delta(t-s)$. The fact that the noise is scaled by $\sqrt{2D}$ is due to the fluctuation dissipation relation. Note that the velocity can be set to $v(q_0) = 0$ by transforming into a co-moving reference frame $x \to x + v(q_0)t$.

Next, define the height field $h(x,t)$ as $\phi(x,t) = \partial_x h(x,t)$ and observe that the equation for $h(x,t)$

$$\partial_t h(x,t) = -\tfrac{1}{2} \mathrm{J}''(q_0) \sqrt{C} (\partial_x h(x,t))^2 + D\partial_x^2 h(x,t) - \sqrt{2D}\xi(x,t) \tag{E.4}$$

is the KPZ equation. The asymptotics of this equation are known exactly [1]

$$\langle \phi(x,t)\phi(0,0) \rangle_\beta^{\mathrm{c}} = \frac{1}{(\lambda_{\mathrm{B}} t)^{2/3}} f_{\mathrm{KPZ}} \left(\frac{x - v(q_0)t}{(\lambda_{\mathrm{B}} t)^{2/3}} \right), \tag{E.5}$$

where $\lambda_{\mathrm{B}} = \sqrt{2C} |\mathrm{J}''(q_0)|$ and $f_{\mathrm{KPZ}}(x)$ is specified in [1, 2]. From this we find

$$\langle q(x,t) q(0,0) \rangle_\beta^{\mathrm{c}} = \frac{C}{(\lambda_{\mathrm{B}} t)^{2/3}} f_{\mathrm{KPZ}} \left(\frac{x - v(q_0)t}{(\lambda_{\mathrm{B}} t)^{2/3}} \right). \tag{E.6}$$

In order to compare this theory with numerical simulations we need to compute the correct normalization C from the microscopic data. This can be done as follows

$$C = \int \mathrm{d}x \, \langle q(x,0) q(0,0) \rangle_\beta^{\mathrm{c}} = \frac{1}{L\Delta x} \left\langle Q^2 \right\rangle_\beta^{\mathrm{c}} = \frac{1}{L\Delta x} \sum_{i,j \text{ odd}} \left\langle q_{i:i+1} q_{j:j+1} \right\rangle_\beta^{\mathrm{c}} \tag{E.7}$$

$$= \frac{1}{L\Delta x} \sum_{i \text{ odd}} \left\langle q_{i:i+1}^2 \right\rangle_\beta^{\mathrm{c}} = \frac{1}{2\Delta x} \left\langle q_{1:2}^2 \right\rangle_\beta^{\mathrm{c}}. \tag{E.8}$$

Here we used $Q = \int \mathrm{d}x\, q(x,0) = \sum_{i \text{ odd}} q_{i:i+1}$ and the translation invariance of the state. Using this we can rewrite Eq. (22) into a prediction after applying τ layers:

$$\langle q_{i:i+1}(\tau\Delta t) q_{1:2}(0)\rangle_\beta^{\mathrm{c}} = \frac{2\Delta x \left\langle q_{1:2}(0)^2\right\rangle_\beta^{\mathrm{c}}}{(\lambda_{\mathrm{B}}\tau\Delta t)^{2/3}} f_{\mathrm{KPZ}}\left(\frac{\Delta x i - v(q_0)\tau\Delta t}{(\lambda_{\mathrm{B}}\tau\Delta t)^{2/3}}\right). \tag{E.9}$$

Using the same units for space and time $\Delta x = \Delta t = 1$, as used throughout the paper, we finally find

$$\langle q_{i:i+1}(\tau) q_{j:j+1}(0)\rangle_\beta^{\mathrm{c}} = \frac{2 \left\langle q_{1:2}(0)^2\right\rangle_\beta^{\mathrm{c}}}{(\lambda_{\mathrm{B}}\tau)^{2/3}} f_{\mathrm{KPZ}}\left(\frac{i - v(q_0)\tau}{(\lambda_{\mathrm{B}}\tau)^{2/3}}\right). \tag{E.10}$$

In particular we find that

$$\max_i \langle q_{i:i+1}(\tau\Delta t) q_{j:j+1}(0)\rangle_\beta^{\mathrm{c}} = \frac{2 \left\langle q_{1:2}(0)^2\right\rangle_\beta^{\mathrm{c}}}{(\lambda_{\mathrm{B}}\tau)^{2/3}} f_{\mathrm{KPZ}}(0), \tag{E.11}$$

with $f_{\mathrm{KPZ}}(0) \approx 0.54$. By fitting this function to the numerically observed maximum of the correlation function as function of τ, we can extract the superdiffusion constant λ_{B}.

F. CONSIDERATIONS ON CIRCUIT $(d,\sigma) = (3, 2312)$

In this section we give further details on the dynamics of $(d,\sigma) = (3, 2312)$, in particular the evolution of an individual $|2\rangle$. This is a good model for $\beta \to \infty$, where only very few $|2\rangle$s are emerged into a background of independently chosen $|0\rangle$s and $|1\rangle$s.

Note that the model is parity symmetric. Hence we can assume the $|2\rangle$ to be initially on an odd site, say $a_1 = 2$. Now consider the case where $a_0 = 1$: If $a_{-1} = 0$ we have the following evolution

$$|\ldots a_{-1}a_0a_1\ldots\rangle = |\ldots 012\ldots\rangle \to |\ldots 020\ldots\rangle \to |\ldots 120\ldots\rangle \to |\ldots 121\ldots\rangle \tag{F.1}$$

and thus after 3 steps we find again the combination $|\ldots 12\ldots\rangle$, but shifted one site to the left. Similarly if $a_{-1} = 1$ we have

$$|\ldots a_{-1}a_0a_1\ldots\rangle = |\ldots 112\ldots\rangle \to |\ldots 120\ldots\rangle\,. \tag{F.2}$$

and thus we find $|\ldots 12\ldots\rangle$ already after 1 step, shifted to the left. Since the probability of $|0\rangle$s and $|1\rangle$s is equal, we therefore find that on average the 2 moves one step to the left every 2 steps, i.e. its velocity is $-1/2$.

Now let us consider the missing case when $a_1 = 2$ and $a_0 = 0$. If $a_2 = 0$ we find

$$|\ldots a_0a_1a_2\ldots\rangle = |\ldots 020\ldots\rangle = |\ldots 120\ldots\rangle \to |\ldots 121\ldots\rangle\,, \tag{F.3}$$

at which point we identify again the 12 combination, implying that the 2 will be a left mover. In the other case $a_2 = 1$ we have

$$|\ldots a_0a_1a_2\ldots\rangle = |\ldots 021\ldots\rangle = |\ldots 121\ldots\rangle\,, \tag{F.4}$$

from which we can read off that the next gate will apply to $|21\rangle$, hence by parity symmetry this $|2\rangle$ will be a right mover. We conclude that single $|2\rangle$'s are either left or right movers with an average velocity $1/2$.

If there are multiple $|2\rangle$s we can say that the total number of left and right movers

$$N_{\mathrm{L}} = \sum_{i \text{ even}} \delta_{a_i 1}\delta_{a_{i+1}2} + \delta_{a_i 0}\delta_{a_{i+1}2}\delta_{a_{i+2}0} + \delta_{a_{i-1}1}\delta_{a_i 2}\delta_{a_{i+1}0} \tag{F.5}$$

$$N_{\mathrm{R}} = \sum_{i \text{ even}} \delta_{a_i 2}\delta_{a_{i+1}1} + \delta_{a_i 0}\delta_{a_{i+1}2}\delta_{a_{i+2}1} + \delta_{a_{i-1}0}\delta_{a_i 2}\delta_{a_{i+1}0} \tag{F.6}$$

are approximately conserved. Under two-body scattering the number of left and right movers seems to be preserved (although we cannot prove it), so that these quantities are only non-conserved by the less frequent three-body (or n-body) scattering events. Therefore, we expect that N_{L} and N_{R} can be treated as approximately conserved for short times, in particular in the low density regime.

G. CONSIDERATION OF GATE WITH A QUASILOCAL DENSITY, $\sigma = 16025$

The gate $(d, \sigma) = (3, 16025)$ has no conserved quantities up to $l, \lambda, \mu = 5$, but has been shown to host a quasi-local conserved quantity in [3], where it is referred to as 'rule 32546187' in their notation. The below figures supports that the manifold of Gibbs state is affected by this quasilocal conserved quantity.

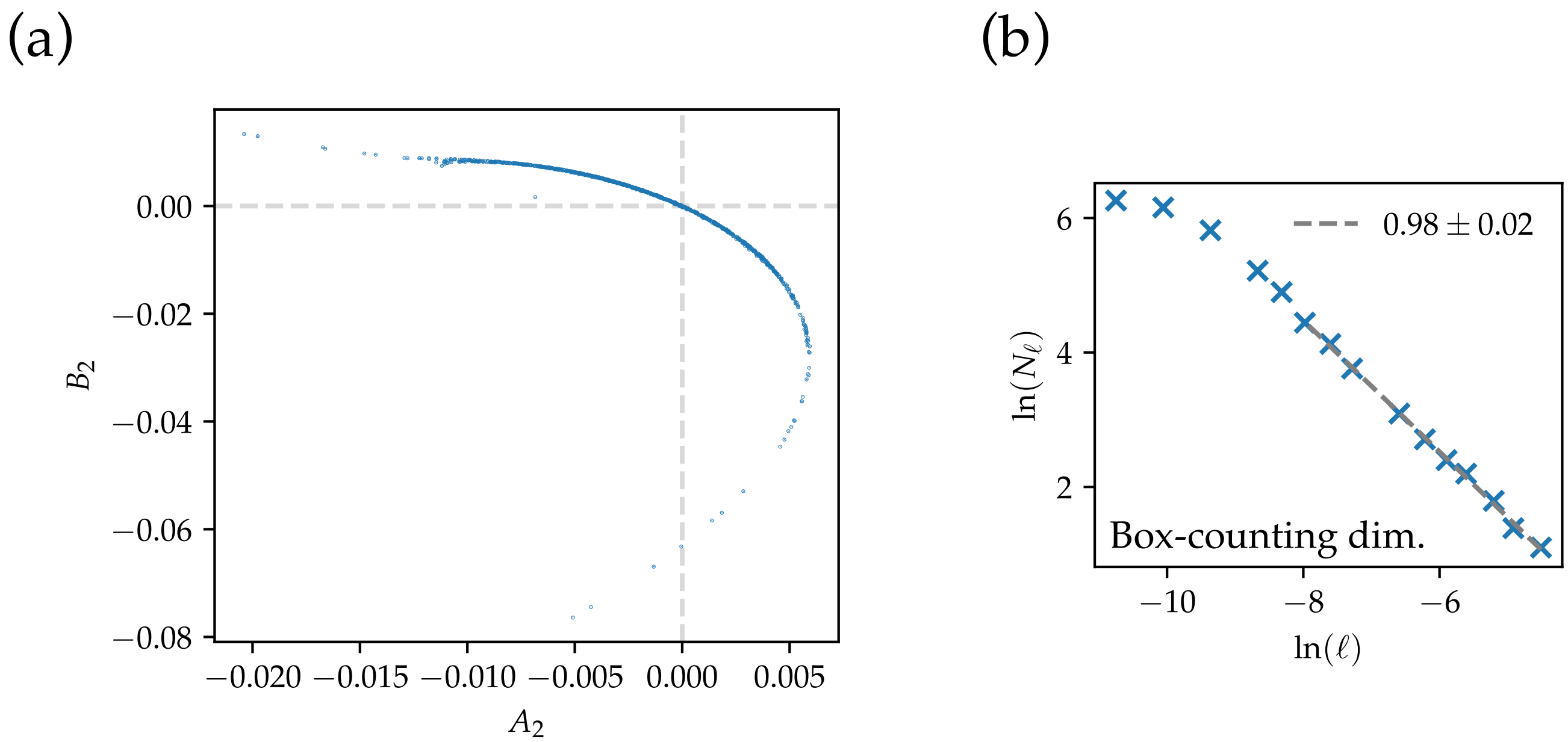

SUPPLEMENTARY FIG. 1. **Manifold of Gibbs states for $(d, \sigma) = (3, 16025)$, a gate with quasilocal conserved quantity. (a)**: Plot of randomly generated observables, displaying a one-dimensional curve. **(b)**: calculating the box-counting dimension. Source data are provided as a Source Data file.

[1] H. Spohn, J. Stat. Phys. **154**, 1191 (2014).
[2] M. Prähofer and H. Spohn, J. Stat. Phys. **115**, 255 (2004).
[3] R. Sharipov, M. Koterle, S. Grozdanov, and T. Prosen, Ergodic behaviors in reversible 3-state cellular automata (2025), arXiv:2503.16593 [cond-mat.stat-mech].
