# Supplementary material for: Circuits as a simple platform for the emergence of hydrodynamics in deterministic chaotic many-body systems
Source: Nat Commun. 2026 Jun 30;17:8152. doi: 10.1038/s41467-026-74457-3 (PMC13458585; doi:10.1038/s41467-026-74457-3)
Supplement: Supplementary file 2 [file 41467_2026_74457_MOESM2_ESM.pdf]

nature portfolio

Peer Review File

Circuits as a simple platform for the emergence of hydrodynamics in deterministic chaotic many-body systems

Corresponding Author: Mr Sun Woo P. Kim

This manuscript has been previously reviewed at another journal. This document only contains information relating to versions considered at Nature Communications.

This file contains all reviewer reports in order by version, followed by all author rebuttals in order by version.

Version 1:

Reviewer comments:

Reviewer #1

(Remarks to the Author)
I thank the authors for their responses. However, as previously communicated, while the paper is scientifically sound and well written and presented, the ideas presented in it are widely known in the community and have been studied and explored before. This remains the main criticism and one the authors have not addressed. They appeal to Hilbert's 6th problem, which is indeed a very interesting problem, but so far there is no clear path towards realizing this program or indeed clues that would warrant such optimism. A very similar points was also made by the other referee. The authors argue that the simplicity of the models will allow them to proceed, but this remains in doubt. Merely presenting the idea, which, as noted repeatedly, has appeared before is not sufficient.

Similarly the authors argue that a verification of predictions of NLFHD in deterministic systems is one of their main achievements. Yet the effective description involves adding a phenomenological noise and a diffusion term, with no hints as to how these emerge microscopically, so that in the end the main achievement is a test of the hydrodynamic equations of NLFHD. But these have been tested before by a number of authors and little new information is gained besides that the postulated effective stochastic hydrodynamic description is correct.

To summarize, the ideas and achievements of the manuscript do not sufficiently advance the field to warrant inclusion in Nature Communications and I do not recommend publication.

Reviewer #3

(Remarks to the Author)
I thank the authors for addressing my questions in the previous version. I think the updated manuscript is suitable for publication in Nature Communications

Open Access This Peer Review File is licensed under a Creative Commons Attribution 4.0 International License, which permits use, sharing, adaptation, distribution and reproduction in any medium or format, as long as you give appropriate credit to the original author(s) and the source, provide a link to the Creative Commons license, and indicate if changes were made.
In cases where reviewers are anonymous, credit should be given to 'Anonymous Referee' and the source.
The images or other third party material in this Peer Review File are included in the article's Creative Commons license, unless indicated otherwise in a credit line to the material. If material is not included in the article's Creative Commons license and your intended use is not permitted by statutory regulation or exceeds the permitted use, you will need to obtain permission directly from the copyright holder.
To view a copy of this license, visit https://creativecommons.org/licenses/by/4.0/

Dear Editor and reviewers of Nature Communications,

Thank you for the comments to our work and the decision made. Here, we would like to reply to the latest reviewer comments.

Best,
Sun Woo P. Kim, Friedrich Hübner, Juan P. Garrahan, Benjamin Doyon

Reviewer #1 (Remarks to the Author):

I thank the authors for their responses. However, as previously communicated, while the paper is scientifically sound and well written and presented, the ideas presented in it are widely known in the community and have been studied and explored before. This remains the main criticism and one the authors have not addressed. They appeal to Hilbert's 6th problem, which is indeed a very interesting problem, but so far there is no clear path towards realizing this program or indeed clues that would warrant such optimism. A very similar points was also made by the other referee. The authors argue that the simplicity of the models will allow them to proceed, but this remains in doubt. Merely presenting the idea, which, as noted repeatedly, has appeared before is not sufficient.

Similarly the authors argue that a verification of predictions of NLFHD in deterministic systems is one of their main achievements. Yet the effective description involves adding a phenomenological noise and a diffusion term, with no hints as to how these emerge microscopically, so that in the end the main achievement is a test of the hydrodynamic equations of NLFHD. But these have been tested before by a number of authors and little new information is gained besides that the postulated effective stochastic hydrodynamic description is correct.

To summarize, the ideas and achievements of the manuscript do not sufficiently advance the field to warrant inclusion in Nature Communications and I do not recommend publication.

Our response:

We have already stated in our previous responses that we are not claiming to have solved Hilbert's 6th Problem. Also we acknowledge(d) that the idea to use circuits to study hydrodynamics was proposed before. However, this was mentioned before either in the context of integrable systems or as an abstract proposal. We fully agree with the reviewer that merely presenting an idea is not sufficient. This is why, as opposed to the other works, we indeed carried out the procedure to derive hydrodynamics for the first time in chaotic circuits.

Our new results include:

- A numerical method to systematically identify the exact analytic form of the conserved quantities
- A numerical method to test that all conserved quantities have been identified. This method also confirms that these circuits relax locally to Generalized Gibbs states. This is a very important observation for hydrodynamics that (1) has not been checked in these systems and (2) also very hard to check in time-continuous systems.

- We identify circuits that have only single site conserved quantities whose hydrodynamics we can compute exactly. Again, there are very few deterministic models available where this can be done, such as integrable systems and anharmonic chains. However, in these systems given as examples, the hydrodynamics is significantly more complicated. On the other hand, in our circuits there are models such as (d, σ) = (3, 996) whose hydrodynamics is equivalent to Burger's equation (arguably the simplest hydrodynamic equation).
- For one of these circuits we numerically establish the validity of the hydrodynamic prediction with significantly higher precision than any previous works known to us. This is an important check that hydrodynamics works in these models and in general. As we showed, some aspects of hydrodynamics converge extremely slowly, such as diffusion for the special point in (3, 996), so this higher precision is essential.
- We check the predictions of NLFHD in these circuits. While we fully agree that NLFHD has been proposed and used before, due to the lack of mode-coupling, here, we can check NLFHD against microscopic simulations with significantly higher precision than any previous works known to us.

We think that this is significantly more than "merely presenting the idea". We agree that we have not rigorously proved the emergence of hydrodynamics in these models. But we find it absurd that the reviewer disqualifies our physical and numerical results, because we are not able to solve one of the largest and most complicated outstanding mathematical problem (Hilbert's 6th problem). Quite on the contrary, given the large amount of work by a broad range of mathematicians and physicists trying to tackle Hilbert's 6th problem, we think it is important to propose new systems where the analysis might be significantly simpler. This is what we do in this paper and also back it up with very precise numerics.

About NLFHD: We do not understand why the reviewer focuses so much on this section. Let us re-iterate that we are not claiming to have rigorously derived the phenomenological hydrodynamic noise, but that our models do not have noise from intrinsic randomness. That was the crux of our very first response. The reviewer agrees with us that the section is scientifically sound. But they say that we only check the predictions of NLFHD numerically (we agree, that is what we do, we never claimed otherwise). Based on this the reviewer argues that we do not sufficiently advance the field. While this individual section contains novel results, we want to remind the reviewer that this is a section of a larger paper built on a new framework we developed and with many further results. Furthermore, the NLFHD check is important for our main message (that hydrodynamics can be more efficiently studied in circuits):

- First, this establishes that NLFHD applies to these circuits as well, which has not been verified before.
- Second, we can check its predictions with large precision because (1) the circuits can be efficiently simulated and (2) because it is only in this single conserved-quantity case that the NLFHD equations have an exact known asymptotic solution. Previous checks compared only to the asymptotic form obtained via the mode-coupling approximation.

Reviewer #3 (Remarks to the Author):

I thank the authors for addressing my questions in the previous version. I think the updated manuscript is suitable for publication in Nature Communications.

Our Response:

Thank you for this comment, we are happy that our changes in the previous response addressed the remaining questions.
